# Key Impact of an Uncommon Plasmid on *Bacillus amyloliquefaciens* subsp. *plantarum* S499 Developmental Traits and Lipopeptide Production

**DOI:** 10.3389/fmicb.2017.00017

**Published:** 2017-01-19

**Authors:** Giulia Molinatto, Laurent Franzil, Sébastien Steels, Gerardo Puopolo, Ilaria Pertot, Marc Ongena

**Affiliations:** ^1^Plant Pathology and Applied Microbiology Unit, Department of Sustainable Agro-Ecosystems and Bioresources, Research and Innovation Centre, Fondazione Edmund MachSan Michele all'Adige, Italy; ^2^Microbial Processes and Interactions Research Unit, Gembloux Agro-Bio Tech Faculty, University of LiègeGembloux, Belgium

**Keywords:** *Bacillus*, genome comparison, plasmid, surfactin, biological control

## Abstract

The rhizobacterium *Bacillus amyloliquefaciens* subsp. *plantarum* S499 (S499) is particularly efficient in terms of the production of cyclic lipopeptides, which are responsible for the high level of plant disease protection provided by this strain. Sequencing of the S499 genome has highlighted genetic differences and similarities with the closely related rhizobacterium *B. amyloliquefaciens* subsp. *plantarum* FZB42 (FZB42). More specifically, a rare 8008 bp plasmid (pS499) harboring a *rap-phr* cassette constitutes a major distinctive element between S499 and FZB42. By curing this plasmid, we demonstrated that its presence is crucial for preserving the typical physiology of S499 cells. Indeed, the growth rate and extracellular proteolytic activity were significantly affected in the cured strain (S499 P^−^). Furthermore, pS499 made a significant contribution to the regulation of cyclic lipopeptide production. Surfactins and fengycins were produced in higher quantities by S499 P^−^, whereas lower amounts of iturins were detected. In line with the increase in surfactin release, bacterial motility improved after curing, whereas the ability to form biofilm was reduced *in vitro*. The antagonistic effect against phytopathogenic fungi was also limited for S499 P^−^, most probably due to the reduction of iturin production. With the exception of this last aspect, S499 P^−^ behavior fell between that of S499 and FZB42, suggesting a role for the plasmid in shaping some of the phenotypic differences observed in the two strains.

## Introduction

Some strains of the *Bacillus amyloliquefaciens* species have been described as beneficial rhizobacteria, because of their ability to promote growth and/or protect plants from infection by multiple pathogens (Lugtenberg and Kamilova, 2009; Cawoy et al., 2011; Kumar et al., 2011). This protective effect against disease is achieved through multiple mechanisms, of which competition for space/nutrients, direct antibiosis against pathogens, and induction of systemic resistance (ISR) in the host plant are the most relevant (Nihorimbere et al., 2011; Bakker et al., 2013). Borriss et al. (2011) separated this bacterial species into two taxa “*B. amyloliquefaciens* subspecies *amyloliquefaciens*” and “*B. amyloliquefaciens* subspecies *plantarum*,” which grouped together all the plant-associated *B. amyloliquefaciens* strains. Recently, the *B. amyloliquefaciens* subspecies *plantarum* has undergone two reclassifications as a later heterotypic synonym of *B. methylotrophicus* (Dunlap et al., 2015), and then as *B. velezensis* (Dunlap et al., 2016). The commercially available strain *B. amyloliquefaciens* FZB42 (FZB42; RhizoVital® 42, Abitep GmbH) is considered as the type strain of this “*plantarum*” subspecies. This is based on its genetic richness in key genes or clusters involved in its plant-associated lifestyle and the synthesis of bioactive secondary metabolites (BSM) acting as signals for intra- or inter-species cross-talks (e.g., stimulation of ISR), and/or as antimicrobials for suppressing competitors in the rhizosphere (Koumoutsi et al., 2004; Chen et al., 2007, 2009; Borriss et al., 2011; Chowdhury et al., 2015).

As part of this BSM arsenal, surfactins, fengycins, and iturins are the three main families of cyclic lipopeptides (CLPs) produced by the *Bacillus amyloliquefaciens* species, synthesized in an mRNA-independent way by modular enzymes (non-ribosomal peptide synthetases, NRPS, or hybrid polyketide synthases/non-ribosomal peptide synthetases, PKS–NRPS; Walsh, 2004). These compounds have multiple functions that are crucial both for rhizosphere fitness of the producing strains but also for their biocontrol potential (Ongena et al., 2005a; Ramarathnam et al., 2007; Romero et al., 2007; Ongena and Jacques, 2008; Kim et al., 2010; Raaijmakers et al., 2010; Cawoy et al., 2015). Surfactins are heptapeptides linked to a β-hydroxy fatty acid (various homologs from C12 to C17) which display some ISR-eliciting activity and some antibacterial and antiviral activity, but are not fungitoxic (Peypoux et al., 1999; Ongena et al., 2007). As wetting agents, surfactins also help the movement of producing cells along the roots by facilitating swarming motility (Kinsinger et al., 2003; Julkowska et al., 2005; Leclère et al., 2006). Moreover, an essential role in the formation of biofilm on roots has been recognized for this CLP (Bais et al., 2004). Fengycins are lipodecapeptides with an internal lactone ring in the peptide moiety, plus a saturated or unsaturated β-hydroxy fatty acid chain (C14–C19), and show strong antifungal activity (Vanittanakom et al., 1986). Iturins include seven variants, of which bacillomycins and mycosubtilin are the best known; all of them are heptapeptides linked to a β-amino fatty acid chain (C14–C17). Iturins have limited antibacterial activity, but a strong antifungal effect (Maget-Dana and Peypoux, 1994). Fengycins and iturins have been shown to be essential for the ISR-independent biocontrol provided by several *Bacillus* strains (Ongena and Jacques, 2008; Raaijmakers et al., 2010).

*Bacillus amyloliquefaciens* subsp. *plantarum* S499 (S499) represents another strain that has been widely investigated due to its biocontrol potential, and more broadly in the context of molecular interaction with the host plant and other soil-borne microorganisms. S499 also synthesizes the three CLP families, but in quite different proportions compared to FZB42 and other *Bacillus amyloliquefaciens* subsp. *plantarum* strains, suggesting a divergent regulatory pathway in the synthesis of secondary metabolites (Cawoy et al., 2015). For instance, S499 is a very efficient producer of surfactins *in vitro* and *in planta*, which correlates with a higher potential for ISR induction compared to FZB42 and other strains belonging to the same subspecies (Jacques et al., 1999; Cawoy et al., 2014). S499 is also quite distinct in terms of other phenotypic traits such as biofilm formation, motility and root colonization (Cawoy et al., 2014). In addition, some environmental factors and plant determinants influence CLP production in S499, making this strain a good model for studying multitrophic interaction in the rhizosphere ecosystem (Nihorimbere et al., 2012; Pertot et al., 2013; Debois et al., 2015). The genome of S499 has recently been sequenced, assembled, and annotated with the scope of pointing out some genetic determinants possibly related to its relatively specific behavior (CP014700–CP014701, Molinatto et al., 2016).

In the work presented here, we performed further comparative genomics, revealing some peculiarities in terms of genetic equipment and organization at chromosome level compared to the type strain FZB42. However, an additional feature of S499 is the presence of a plasmid (pS499) containing a *rap-phr* cassette encoding the response regulator aspartate phosphatase (Rap) and its putative Rap regulatory peptide (Phr). As Rap-Phr systems have pleiotropic regulatory effects on a number of cellular processes (Pottathil and Lazazzera, 2003), we pursued the functional characterisation of pS499 by evaluating how its loss affects S499 behavior compared to the type strain FZB42. Our data provide some evidence regarding the crucial impact of this plasmid on several traits related to rhizosphere competence, such as substrate utilization, cell motility, biofilm formation, and antagonism against fungal phytopathogens.

## Materials and methods

### Bacterial and fungal strains

All the bacterial strains used in this work (Table S1) were stored at length in glycerol 30% at −80°C and routinely grown at 28°C in Luria-Bertani broth (LB; tryptone 10 g l^−1^, yeast extract 5 g l^−1^, NaCl 10 g l^−1^, pH 7) and on LB broth amended with agar 16 g l^−1^ (LBA). The phytopathogenic fungi used in this work (Table S1) were grown on potato dextrose agar (PDA) at 28°C and stored at length on PDA slants at room temperature.

### Genome comparative analysis

Phylogenetic analysis was carried out with Gegenees software 2.2.1 (Ågren et al., 2012) through fragmented all-against-all comparison (fragment size = 500; sliding step size = 500) performed on the sequences of *B. amyloliquefaciens* subsp. *plantarum* and closely related strains whose complete genome was present on NCBI (http://www.ncbi.nlm.nih.gov) on 14 March 2016. In particular, 19 strains belonging to different *Bacillus* species were included in the analysis. The heat plot tab generated by Gegenees was used to build the Neighbor-Joining phylogenetic tree with the Neighbor and DrawGram applications of the Phylogeny Inference Package (PHYLIP) version 3.695 (Felsenstein, 1989).

Genome alignment of the S499 genome against FZB42 was done with the sequence-based comparison tool on SEED Viewer version 2.0 (Overbeek et al., 2005). On the same platform, the BLAST tool was used to perform gene by gene sequence alignments. The genomes of FZB42 and S499 were also aligned by applying the progressive algorithm and maintaining the default settings implemented in open-source MAUVE aligner v2.3.1 (Darling et al., 2010). BRIG application (Alikhan et al., 2011) was used to display circular comparisons.

To assess the frequency of plasmids similar to the S499 plasmid (pS499), the NCBI Genome Assembly, and Annotation reports for *B. amyloliquefaciens* subsp. *plantarum* and *B. amyloliquefaciens* subsp. *amyloliquefaciens*, and the NCBI Plasmid Annotation report for *B. subtilis* were examined (included sequences are listed in Table S2). Moreover, strains 23, 76, 98R, 98S, 104, and GA1 belonging to different *Bacillus* spp. were tested using PCR with Rap1 primers (Table S3) after isolation of plasmid DNA using a GeneJET Plasmid Miniprep kit (Thermo Fisher Scientific Inc.). The PCR program was as follows: 5 min at 95°C, followed by 30 cycles of 30 s at 95°C, 30 s at 60°C and 1 min at 68°C. Annotation of the pS499 sequence (CP014701) was done using Prokka (Seemann, 2014). The sequence encoding the Phr peptide was retrieved by aligning the *phrQ* gene (BAPNAU_RS20550, Wu et al., 2013) against the pS499 sequence. Alignments of the plasmid-encoded *rap* genes were performed on NCBI blastn suite (http://blast.ncbi.nlm.nih.gov/Blast.cgi) and with EMBOSS Needle Pairwise Sequence Alignment tools (http://www.ebi.ac.uk/Tools/psa/).

### Plasmid curing

*Bacillus amyloliquefaciens* subsp. *plantarum* S499 was cured of its native plasmid according to the procedure described by Feng et al. (2013), with some modifications. Briefly, S499 was grown for 16 h at 30°C (180 rpm) in sterile 15 ml tubes containing 5 ml of LB broth. The resulting cell culture was diluted 10 times in LB broth and 50 μl were transferred to sterile 15 ml tubes containing 5 ml of LB broth amended with 0.005% sodium dodecyl sulfate. Inoculated tubes were incubated at 42°C (180 rpm) for 12 h. This step was repeated 14 times. After each 12 h incubation, serial dilutions of the cell cultures were streaked on LBA dishes and incubated at 30°C for 24 h. Selected colonies were picked up and total DNA was extracted using a PureLink Genomic DNA Mini Kit (Thermo Fisher Scientific Inc.) according to the manufacturer's instructions. The extracted total DNA was amplified in PCR reactions, where Rep primers (Table S3) specific for the pS499 sequence encoding *rep* gene were used. To rule out the possibility that potential plasmid cured derivatives were the result of contaminations, 16S rDNA, *cheA, gyrA* genes from S499 and its derivatives were amplified using primer pairs reported in Table S3. In all the cases, the PCR programs consisted of a first step at 95°C for 5 min and 30 cycles in series of 30 s at 95°C, 30 s at 60°C, 1 min at 72°C and finally 5 min at 72°C. The presence/absence of the *rep* amplicons was checked on a 1% agarose gel to select plasmid cured derivatives. Amplicons from 16S rDNA, *cheA, gyrA* genes were purified with illustra ExoProStar 1-Step (GE Healthcare Europe GmbH), and sequenced with an ABI PRISM 3730xl DNA analyzer (Applied Biosystems, Thermo Fisher Scientific Inc., USA). To determine the level of nucleotide sequence identity, sequences of 16S rDNA, *cheA, gyrA* amplicons deriving from S499 and its derivative were subsequently aligned with EMBOSS Needle Pairwise Sequence Alignment tools (http://www.ebi.ac.uk/Tools/psa/).

### Growth curves

A SpectraMax M2E Multi-Mode Microplate Reader (Molecular Devices LLC, USA) was used to determine the growth rates of S499, and the plasmid-cured S499 strain (S499 P^−^). FZB42 was included in all the following assays as a comparison. The test was carried out in sterile 48-well plates. A volume of 10 μl of a cell suspension [optical density at 600 nm (OD_600_) = 0.001 corresponding to the 10^3^ colony forming units (CFU)] was inoculated into LB broth (1 ml) and a modified LB broth (1 ml), where tryptone (10 g l^−1^) was replaced by casamino acids (10 g l^−1^). Non-inoculated LB and modified LB broths were used as a control. The plate was incubated with continuous shaking for 40 h at 28°C and OD_600_ was measured every 30 min. Three wells were used for each strain (replicates) and the experiment was repeated.

The supernatants of the 48-well LB cultures were filtered through a 0.2 μm membrane (Sartorius AG, Germany) at the end of the incubation period (40 h). To identify and quantify CLPs (surfactins, fengycins, and iturins), the culture filtrates of each strain were analyzed with ultra-performance liquid chromatography—electrospray ionization mass spectrometry (UPLC-ESI-MS) according to the procedure described below. Three repetitions of the assay were used to calculate the mean values of production.

### Extracellular proteolytic activity

The FZB42, S499 and S499 P^−^ strains were inoculated into 15 ml sterile tubes containing 5 ml of LB broth and grown overnight (16 h) at 28°C (180 rpm). The resulting cell cultures were diluted 100 times by transferring 50 μl into sterile 15 ml tubes containing 5 ml of LB broth (three replicates for each strain). Once inoculated, 15 ml tubes were incubated for 6 h in the same conditions reported above. OD_600_ was recorded at the end of the incubation period. Subsequently, tubes were centrifuged at 4000 rpm for 10 min to remove the cells and the supernatants were filtered through a 0.2 μm membrane (Sartorius AG). A volume of 225 μl of culture filtrates (three replicates for each tube) was transferred to 1.5 ml sterile microfuge tubes and mixed with 150 μl of 1% azocasein stock solution (50 mM Tris-HCl, pH 8.8). After 4 h of incubation at 37°C, the undigested substrate was precipitated by adding 375 μl of 5% trichloroacetic acid and centrifuged for 3 min at 13,200 rpm. Supernatants were transferred to new 1.5 ml sterile microfuge tubes containing 400 μl of 1 M NaOH, and absorbance at 405 nm (OD_405_) was then recorded. Relative proteolytic activity was calculated as the ratio of the OD_405_/OD_600_. The experiment was repeated.

The production of extracellular proteases was also assessed on skimmed milk dishes. For this purpose, a volume of 5 μl of a cell suspension (OD_600_ = 1) corresponding to 5 × 10^5^ CFU for each strain was inoculated onto LBA amended with 1% (w/v) of skimmed milk. Once inoculated, Petri dishes were incubated at 28°C and the diameter of the clarification halo was measured after 48 h. Five Petri dishes (replicates) were used for each strain and the experiment was repeated.

### Kinetics of cyclic lipopeptide production

The FZB42, S499, and S499 P^−^ strains were grown overnight on LBA at 30°C in Petri dishes. Bacterial cells were scraped from LBA surface and collected with a loop in 1 ml of sterile distilled H_2_O, washed three times in sterile distilled H_2_O, and the OD_600_ was adjusted to 1. A volume of 1 ml of the deriving cell suspensions was inoculated into Erlenmeyer flasks containing 100 ml of LB broth. Flasks were then incubated for 24 h at 28°C (110 rpm). The samples were collected every hour from 0 to 12 h with final sampling after 24 h. From each flask, 3 ml of cell culture were transferred to three microfuge tubes (1 ml each), which were centrifuged for 5 min at 12,600 rpm. Supernatants were used for the identification and quantification of cyclic lipopeptides with UPLC-ESI-MS. Cell pellets were stored at −20°C for analysis of gene expression (*srfA* and *rap*) using RNA extraction and quantitative reverse transcription-polymerase chain reaction (qRT-PCR), and measurement of bacterial growth with flow cytometry. The experiment was carried out three times.

Samples were analyzed using reverse phase UPLC (Acquity class H, Waters Corp., USA) coupled with a single quadrupole MS (SQ Detector, Waters Corp.) on an Acquity UPLC BEH C18 2.1 × 50 mm, 1.7 μm column (Waters Corp.). Elution started at 30% acetonitrile (flow rate of 0.60 ml min^−1^). After 2.43 min, the percentage of acetonitrile was brought up to 95% and held for 5.2 min. Then, the column was stabilized at 30% acetonitrile for 1.7 min. Compounds were identified based on their retention times compared to authentic standards (98% purity; Lipofabrik Society, France) and the masses detected in the SQDetector. Ionization and source conditions were set as follows: source temperature = 130°C; desolvation temperature = 400°C; nitrogen flow = 1000 l h^−1^; cone voltage = 120 V.

### Quantification of relative gene expression

Total RNA was extracted with a NucleoSpin® RNA kit (Macherey-Nagel GmbH & Co. KG, Germany) according to the manufacturer's instructions. Quantification of *srfA* and pS499 *rap* relative gene expression was done using reverse transcription Real-time PCR (StepOnePlus™, Thermo Fisher Scientific Inc.), with a qPCRBIO SyGreen 1-Step Hi-ROX kit (PCR Biosystems Ltd, UK). Primers used in the reactions were designed by Primer3web version 4.0.0 (Untergasser et al., 2012) and are listed in Table S3. The qRT-PCR program consisted of a first step of 10 min at 48°C, followed by 2 min at 95°C, and 40 cycles in series of 5 s at 95°C and 30 s at 60°C. The housekeeping gene *gyrA* was used as an endogenous control.

The relative gene expression was calculated according to the comparative C_T_ method (Livak and Schmittgen, 2001). At each time point, ΔC_T_ was determined by subtracting the threshold cycle (C_T_) value of *gyrA* from the C_T_ value of the target gene; then the ΔC_T_ of sample “time 0” was subtracted from the ΔC_T_ values of the following sampling times, obtaining ΔΔC_T_ values. Finally, the relative quantity (RQ) of gene expression was calculated according to this formula:

$$\begin{array}{l} \text{RQ}=2^{-\Delta \Delta \text{CT}} \end{array}$$

### Measurement of bacterial growth with flow cytometry

A first sonication step was used to dissolve cellular aggregates. Cell pellets were suspended in 500 μl of staining solution S1 (72 g l^−1^ tartaric acid; 3.89 g l^−1^ Na_2_HPO_4_; 2.85 g l^−1^ EDTA; 0.0375 g l^−1^ sucrose monohydrate; pH 3) over three cycles of gentle sonication (15–20 s at 25–30% of the power of the device, Sonopuls HD 2070, Bandelin GmbH, Germany). Subsequently, 500 μl of solution S2 (1.95 g l^−1^ citric acid; 5.8 g l^−1^ NaCl; 1.45 g l^−1^ Na_2_HPO_4_; pH 3.8) supplied with 0.2% acridine orange were added. Samples were then mixed by vortexing thoroughly, before being analyzed with flow cytometry (BD Accuri™ C6, Becton, Dickinson and Company, USA) for cell counting.

### Swarming motility and biofilm formation

The swarming motility of FZB42, S499, and S499 P^−^ was evaluated according to Pertot et al. (2013). The diameter of the bacterial macrocolonies was measured at 12, 16, and 20 h after inoculation. Four Petri dishes (replicates) were used for each strain and the experiment was repeated.

The production of biofilm by FZB42, S499, and S499 P^−^ in 24-well polystyrene plates was determined according to Pertot et al. (2013). Specific biofilm formation (SBF) was calculated according to Yaryura et al. (2008) with the following formula:

$$\begin{array}{l} \text{SBF}=\left(\text{B}-\text{NC}\right)/\text{BG} \end{array}$$

where B is the amount of crystal violet attached to the well surfaces measured at 590 nm (OD_590_), NC is the OD_590_ of the negative control and BG is the bacterial growth measured at 600 nm (OD_600_). Eight wells (replicates) were used for each strain and the experiment was repeated.

### Antagonism against fungal phytopathogens

The antifungal activity of FZB42, S499, and S499 P^−^ was tested against *Cladosporium cucumerinum* and *Fusarium oxysporum* f. sp. *radicis-lycopersici*. *Cladosporium cucumerinum* was streaked over the whole LBA surface in Petri dishes, and subsequently a volume of 5 μl of bacterial cell suspension (OD_600_ = 1) corresponding to 5 × 10^5^ CFU was inoculated onto it. Petri dishes were incubated at 28°C for 72 h. To test for *F. oxysporum* f. sp. *radicis-lycopersici*, bacterial cells were inoculated onto LBA, 2 cm from the edge of the Petri dishes. Once inoculated, dishes were incubated at 28°C for 72 h. Plugs of *F. oxysporum* f. sp. *radicis-lycopersici* mycelium (5 mm) were cut away from the edge of young (5-day-old) colonies grown on PDA and placed 2.5 cm from the bacterial colonies. Petri dishes were incubated at 28°C for 72 h. Dishes not inoculated with the bacterial strains were used as a control. At the end of the incubation period, the inhibition zone (distance between bacterial colonies and mycelia) was measured. Moreover, two plugs (5 mm) of medium were removed from the inhibition zone and transferred to 1.5 ml microfuge tubes containing 1 ml of 50% acetonitrile and 0.1% formic acid. Cyclic lipopeptides were extracted by regular vortexing for 2 h at room temperature. Then, samples were centrifuged and filtered through a 0.2 μm membrane (Sartorius AG) before being injected into UPLC-ESI-MS columns according to the procedure reported above. Three dishes were used for each combination (replicates) and the experiment was repeated.

### Statistical analysis

As the *F*-test (α = 0.05) revealed non-significant differences between repeated experiments (*p* > 0.5), the data were pooled. For proteolytic activity, CLP production, swarming motility, and biofilm formation assays, data were subjected to one-way ANOVA. The data obtained in the antagonism experiments were subjected to multifactorial ANOVA. Tukey's test (α = 0.05) was used to perform mean pairwise comparisons. Statistical analysis was carried out using Excel (Microsoft Corp., USA) and Statistica (Dell Inc., USA).

## Results

### Specific genetic traits of the *Bacillus amyloliquefaciens* subsp. *plantarum* S499 chromosome

**Figure 1:**
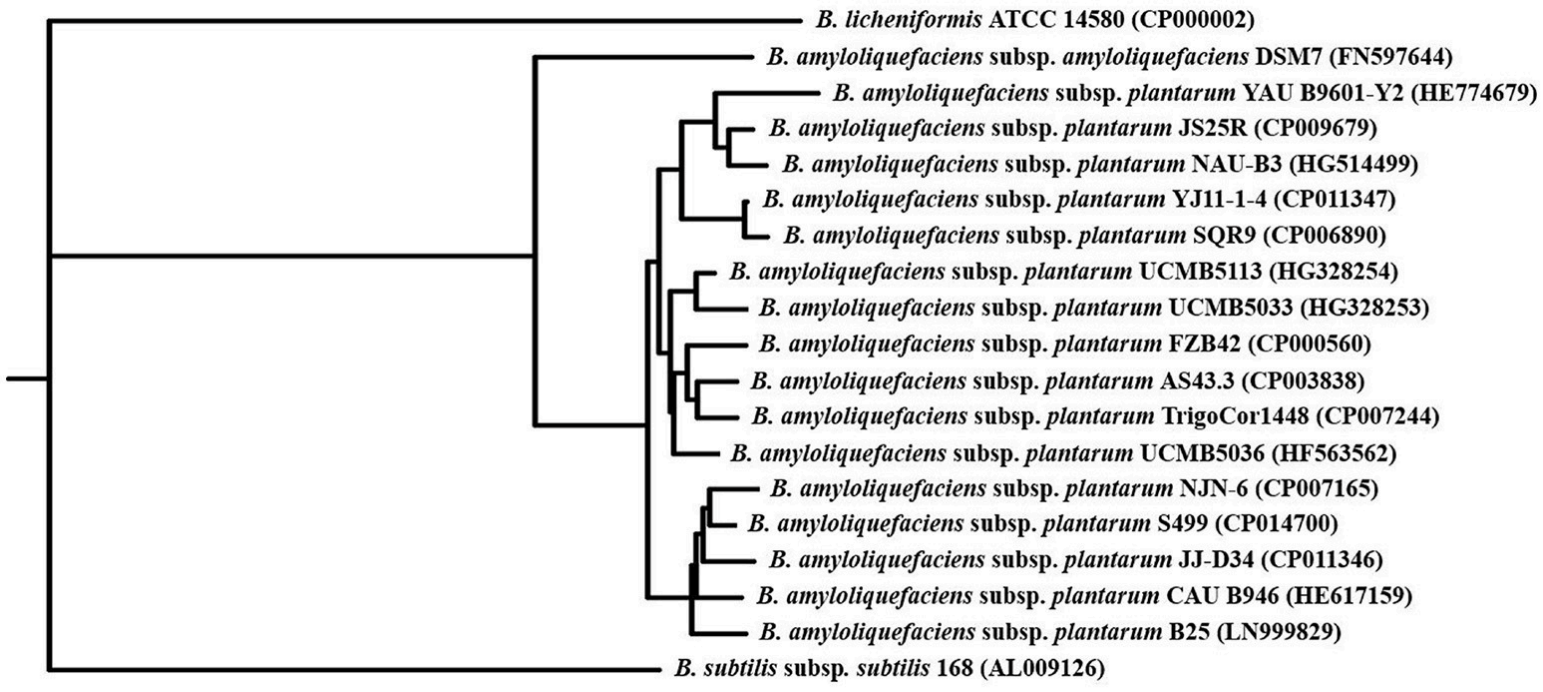
**Phylogenetic tree of ***Bacillus amyloliquefaciens*** subsp. ***plantarum*****. The Neighbor-Joining phylogenetic tree was obtained with PHYLIP applications after fragmented all-against-all comparison performed using Gegenees software 2.2.1 with the complete genome sequences of 19 strains belonging to the *Bacillus* genus (accession numbers are reported in brackets).

Comparative genomics first confirmed that S499 belongs to the taxonomic group of the *B. amyloliquefaciens* subsp. *plantarum*. Indeed, its genome shares the highest level of sequence similarity with the NJN-6, JJ-D34, CAU B946, and B25 strains, forming a subclade of the branch including FZB42 and all other strains classified under the “*plantarum*” subspecies. This branch is separated from those of the closely related *B. amyloliquefaciens* subsp. *amyloliquefaciens* DSM7, *B. subtilis* subsp. *subtilis* 168, and *B. licheniformis* ATCC 14580 (Figure 1). However, comparison of general genomic features (genome size, G+C content, the number of coding sequences, RNA operons, tRNAs, and insertion sequence elements) showed high similarity with these species. A significant difference emerged in the content of phage-related genes. Indeed, the S499 genome has 154 phage-related genes, while only 44 and 71 of these genes are present in the FZB42 and *B. licheniformis* ATCC 14580 genomes, respectively. However, the phage-related gene content in the S499 genome is lower than that of *B. amyloliquefaciens* subsp. *amyloliquefaciens* DSM7 (273 genes) and *B. subtilis* subsp. *subtilis* 168 (268 genes) (Table 1).

**Table 1:** **Principal genomic features of ***Bacillus amyloliquefaciens*** subsp. ***plantarum*** S499, in comparison with the genomes of the closely related ***Bacillus*** spp**.

	***B. amyloliquefaciens* subsp. *plantarum* S499**	***B. amyloliquefaciens* subsp. *plantarum* FZB42**	***B. amyloliquefaciens* subsp. *amyloliquefaciens* DSM7**	***B. subtilis* subsp. *subtilis* 168**	***B. licheniformis* ATCC 14580**
Genome size (bp)	3,927,922	3,918,589	3,980,199	4,214,814	4,222,645
G+C contents (%)	46.6	46.4	46.1	43.5	46.2
Coding sequences (CDS)	3974	3863	3924	4114	4199
Ribosomal RNA operons	8	10	10	10	7
Number of tRNAs	81	89	94	86	72
Plasmids	1	–	–	–	–
Insertion sequence elements	1	9	18	–	10
Phage-associated genes	154	44	273	268	71

**Figure 2:**
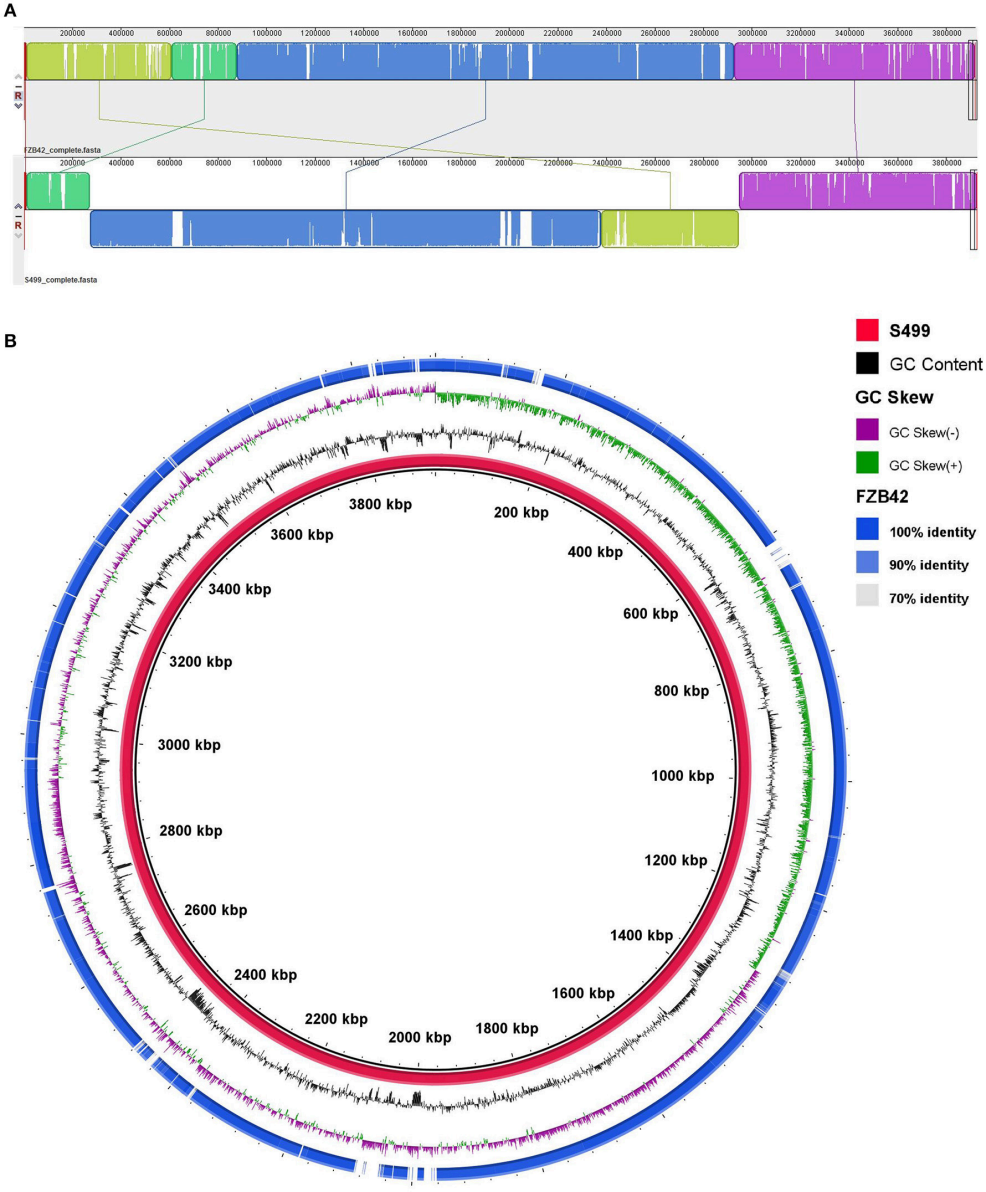
**Whole-genome comparison between the ***Bacillus amyloliquefaciens*** subsp. ***plantarum*** S499 and ***Bacillus amyloliquefaciens*** subsp. ***plantarum*** FZB42 genomes**. **(A)** MAUVE alignment shows genomic rearrangement of the S499 genome compared to the FZB42 chromosome. **(B)** BRIG alignment of the S499 and FZB42 genomes. From inner to outer ring: (1) nucleotide sequence of the S499 genome; (2) GC percent; (3) GC skew; (4) Blast comparison with the FZB42 genome.

Assembly of the S499 genome sequence revealed that its chromosome has a different arrangement, including a large inversion compared to the FZB42 genome (Figure 2A). Alignment against the FZB42 genome showed 98% of nucleotide identity out of 94% query cover (Figure 2B). The S499 genome shares 46 and 88% of coding sequences (CDS), with respectively >99% and >90% identity at amino acid level with the FZB42 genome. Their core genomes include 3547 CDS. As regards homologous genes with functions related to rhizosphere competence, plant-growth promotion and antimicrobial activity, nucleotide identity is always above 95%, with one case of 100% sequence identity for the *abrB* gene (Table 2).

**Table 2:** **Genes involved in root colonization, plant growth promotion and biocontrol common to ***Bacillus amyloliquefaciens*** subsp. ***plantarum*** S499 and ***Bacillus amyloliquefaciens*** subsp. ***plantarum*** FZB42 (sequence similarity is expressed as the percentage of nucleotide identity)**.

**Gene name**	**Locus tag**		**Product**	**Identity (%)**
	**S499**	**FZB42**		
**MOTILITY AND BIOFILM FORMATION**				
*abrB*	AS588_RS09635	RBAM_RS05945	Transition state regulator	100
*ecsA*	AS588_RS10440	RBAM_RS05140	ABC transporter ATP-binding protein	98
*ecsB*	AS588_RS10435	RBAM_RS05145	ABC transporter permease	98
*ecsC*	AS588_RS10430	RBAM_RS05150	ABC transporter-associated protein	98
*Efp*	AS588_RS04045	RBAM_RS11340	Elongation factor P	98
*epsA-O*	AS588_RS15840-AS588_RS15910	RBAM_RS15740-RBAM_RS15810	Operon for capsular polysaccharides biosynthesis	96–98
*degU*	AS588_RS16430	RBAM_RS16295	Two-component response regulator	99
*fla-che*	AS588_RS07105-AS588_RS07250	RBAM_RS07995-RBAM_RS08140	Operon for flagellar synthesis and chemotaxis	98–99
*lytS*	AS588_RS02230	RBAM_RS12940	Sensor histidine kinase	98
*motA*	AS588_RS08495	RBAM_RS06710	Flagellar motor rotation protein	98
*motB*	AS588_RS08500	RBAM_RS06705	Flagellar motor rotation protein	98
*pgsA-C*	AS588_RS16615-AS588_RS16625	RBAM_RS16480-RBAM_RS16490	Operon for poly-γ-glutamate synthesis	99
*resE*	AS588_RS10795	RBAM_RS04785	Sensor histidine kinase	98
*sacB*	AS588_RS17695	RBAM_RS17620	Levan sucrose	98
*Sfp*	AS588_RS12395	RBAM_RS01880	Phosphopantetheinyl transferase necessary for surfactin synthesis	98
*sigH*	AS588_RS13505	RBAM_RS00625	Sigma factor H	98
*sigW*	AS588_RS13075	RBAM_RS01130	ECF sigma factor W	99
*sinI*	AS588_RS03975	RBAM_RS11410	SinR antagonist	99
*sinR*	AS588_RS03970	RBAM_RS11415	Master regulator of biofilm formation	99
*spo0A*	AS588_RS04160	RBAM_RS11225	Master regulator of initiation of sporulation	98
*srfABCD*	AS588_RS12405-AS588_RS12420	RBAM_RS01840-RBAM_RS01855	Surfactin synthetases	97–98
*swrA*	AS588_RS16295	RBAM_RS16170	Swarming protein	95
*swrB*	AS588_RS07095	RBAM_RS08150	Swarming protein	96
*swrC*	AS588_RS00450	RBAM_RS03555	Multidrug efflux pump	98
*yqxM-sipW-tasA*	AS588_RS03955-AS588_RS03965	RBAM_RS11420-RBAM_RS11430	Operon essential for biofilm formation	97–98
*ycbA*	AS588_RS12820	RBAM_RS01415	Sensor histidine kinase	96
*ycdH*	AS588_RS12675	RBAM_RS01565	High affinity zinc ABC transporter lipoprotein	98
*yfiQ*	AS588_RS01060	RBAM_RS04220	Putative surface adhesion protein	98
*ylbF*	AS588_RS07835	RBAM_RS07405	Positive regulator of ComK	99
*ymcA*	AS588_RS06825	RBAM_RS08420	Protein involved in community development	99
*yqeK*	AS588_RS03495	RBAM_RS11910	Putative HD phosphatase	98
*yusV*	AS588_RS15075	RBAM_RS15000	Protein involved in swarming/biofilm formation	98
**CARBOHYDRATE CATABOLISM**				
*abnA*	AS588_RS02280	RBAM_RS12890	Endo 1,5-alpha-L-arabinase	98
*bglC*	AS588_RS06260	RBAM_RS09035	Endo-1,4-beta-glucanase	96
*bglS*	AS588_RS18110	RBAM_RS18065	Endo-beta-1,3-1,4 glucanase	97
*eglS*	AS588_RS06260	RBAM_RS09035	Endo-1,4-beta-glucanase	96
*galE1*	AS588_RS09290	RBAM_RS06070	UDP-glucose 4-epimerase	97
*galK1*	AS588_RS09285	RBAM_RS06075	Galactokinase	97
*galT1*	AS588_RS09295	RBAM_RS06065	Galactose-1-phosphate uridyltransferase	97
*ganA*	AS588_RS09300	RBAM_RS06060	Arabinogalactan endo-1,4-beta-galactosidase	97
*kdgA*	AS588_RS06305	RBAM_RS08975	2-dehydro-3-deoxyphosphogluconate aldolase	98
*kdgK*	AS588_RS06315	RBAM_RS08965	2-dehydro-3-deoxygluconate kinase	97
*lacE*	AS588_RS09280	RBAM_RS06080	Phosphotransferase system	97
*lacF*	AS588_RS09275	RBAM_RS06085	Phosphotransferase system	99
*lacG*	AS588_RS09270	RBAM_RS06090	Putative 6-phospho-beta-galactosidase	98
*lacR*	AS588_RS09265	RBAM_RS06095	Lactose phosphotransferase system repressor protein	97
*Pel*	AS588_RS00695	RBAM_RS03860	Pectate lyase	97
*pelB*	AS588_RS18195	RBAM_RS18130	Pectin lyase	97
*Pgm*	AS588_RS09260	RBAM_RS06100	Predicted phosphoglucomutase	97
*yhfE*	AS588_RS10370	RBAM_RS05210	Glucanase/aminopeptidase	99
*ysdC*	AS588_RS02275	RBAM_RS12895	Putative endo-1,4-beta-glucanase	98
*xylA*	AS588_RS06580	RBAM_RS08680	Xylose isomerase	97
*xylB*	AS588_RS06575	RBAM_RS08685	Xylulose kinase	97
*xynA*	AS588_RS17000	RBAM_RS16860	Xylanase	96
*xynD*	AS588_RS06235	RBAM_RS09060	Xylanase	97
*xynP*	AS588_RS06595	RBAM_RS08665	Hypothetical symporter of oligosaccharides	97
*xynB*	AS588_RS06590	RBAM_RS08670	Xylan beta-1,4-xylosidase	96
**PLANT GROWTH PROMOTION**				
*alsD*	AS588_RS16685	RBAM_RS16550	Acetolactate decarboxylase	98
*alsS*	AS588_RS16690	RBAM_RS16555	Acetolactate synthase	99
*alsR*	AS588_RS16695	RBAM_RS16560	LysR transcriptional regulator	98
*bdhA*	AS588_RS00220	RBAM_RS03320	2,3-butanediol dehydrogenase	99
*dhaS*	AS588_RS05800	RBAM_RS09505	Putative indole-3-acet-aldehyde dehydrogenase	98
*Phy*	S588_RS05510	RBAM_RS09795	Phytase	98
*yhcX*	AS588_RS10830	RBAM_RS04750	Nitrilase	98
*ysnE*	AS588_RS17725	RBAM_RS17660	Putative IAA acetyl-transferase	96
*ywkB*	AS588_RS17210	RBAM_RS17070	Putative auxin efflux carrier	97
**ANTIMICRIOBAL ACTIVITY (GENE CLUSTERS FOR NON-RIBOSOMALLY SYNTHESIZED PEPTIDES AND POLYKETIDES)**				
*bacA-E*	AS588_RS17515-AS588_RS17535	RBAM_RS17420-RBAM_RS17440	Bacilysin	97–99
*baeB-S*	AS588_RS06740-AS588_RS06800	RBAM_RS08445-RBAM_RS08505	Bacillaene	97–98
*bmyA-D*	AS588_RS06215-AS588_RS06230	RBAM_RS09065-RBAM_RS09080	Iturin	96–97
*dfnA-M*	AS588_RS04400-AS588_RS04470	RBAM_RS10910-RBAM_RS10980	Difficidin	96–98
*dhbA-F*	AS588_RS14595-AS588_RS14615	RBAM_RS14490-RBAM_RS14510	Bacillibactin	96–97
*fenA-E*	AS588_RS06080-AS588_RS06100	RBAM_RS09195-RBAM_RS09215	Fengycin	96
*mlnA-I*	AS588_RS08035-AS588_RS08075	RBAM_RS07160-RBAM_RS07200	Macrolactin	96–97
*srfA-D*	AS588_RS12405-AS588_RS12420	RBAM_RS01840-RBAM_RS01855	Surfactin	97–98

A large number (46%) of S499 CDS not shared with FZB42 are annotated as hypothetical proteins, whereas 30% are phage-related genes. Moreover, genes putatively involved in primary metabolism (10%), transcriptional regulation (4%), transport systems (2%), tetracycline resistance, and lanthionine biosynthesis are included in the remaining unique S499 CDS. Similarly to S499, a considerable number of FZB42 unique CDS (44%) are also classified as hypothetical proteins. However, among function-annotated CDS there are ribosomal genes (12%), genes encoding enzymes involved in transport, and detoxification (6%), genes related to restriction systems (5%), transcriptional regulation (3%), and acriflavine resistance (Table S4).

### A rare plasmid is present in the genome of *Bacillus amyloliquefaciens* subsp. *plantarum* S499

**Figure 3:**
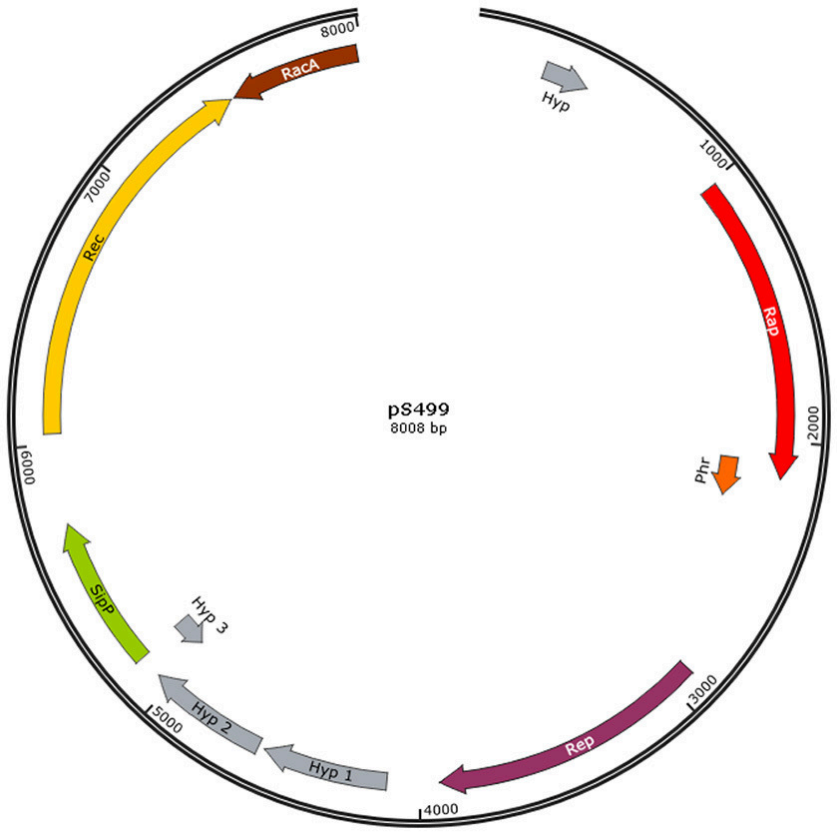
**Graphic representation of plasmid pS499 from ***Bacillus amyloliquefaciens*** subsp. ***plantarum*** S499**. Structure of plasmid pS499 according to PROKKA annotations (Hyp, hypothetical protein; Rap, response regulator aspartate phosphatase; Phr, phosphate regulator peptide; Rep, replication protein; SipP, signal peptidase I P; Rec, recombination protein; RacA, chromosome-anchoring protein).

An additional important genetic feature distinguishing S499 from FZB42 is the presence of plasmid DNA (8008 bp). Based on the plasmid:chromosome sequence coverage ratio, we estimate that there are at least two copies of this plasmid (pS499) per cell. The few genes located on pS499 (Figure 3) encode proteins involved in replication (AS588_19065, Rep) and mobilization (AS588_19090, Rac; AS588_19095, RecA) of the plasmid, a signal peptidase (AS588_19085, SipP), and a response regulator aspartate phosphatase (AS588_19060, Rap). Moreover, we identified a plasmid region encoding the putative Rap regulatory peptide (Phr) located downstream of the *rap* gene. In addition to this plasmid *rap-phr* cassette, nine genes encoding different members of the Rap family are located on the chromosome of S499. Seven of these genes show 98–99% nucleotide identity with the homologous genes of FZB42 (Table 3).

**Table 3:** **Genes encoding Rap proteins identified in the genome of ***Bacillus amyloliquefaciens*** subsp. ***plantarum*** S499 and sequence similarity with the homologous genes of ***Bacillus amyloliquefaciens*** subsp. ***plantarum*** FZB42**.

**Gene name**	**Locus tag**		**Product**	**Identity at nucleotide level (%)**
	**S499**	**FZB42**		
**CHROMOSOME**				
*rapA_2*	AS588_RS09075	RBAM_RS06230	RapA	99
*rapI_1*	AS588_RS11695	–	RapI	–
*rapF_1*	AS588_RS12260	RBAM_RS02015	RapF/RapC	99
*rapJ*	AS588_RS12690	RBAM_RS01550	RapJ	99
*rapH*	AS588_RS14175	RBAM_RS02305	RapH	53
*rapF_2*	AS588_RS16895	RBAM_RS16755	RapF	98
*rapA_4*	AS588_RS17040	RBAM_RS16900	RapA/RapB	99
*rapF_3*	AS588_RS17360	RBAM_RS17235	RapF	98
*rapI_3*	AS588_RS18595	RBAM_RS18570	RapI/RapX	98
**PLASMID**				
*rapA_5*	AS588_19060	–	RapA/RapQ	–

Comparison of the 32 available genome sequences of other *B. amyloliquefaciens* subsp. *plantarum* strains revealed that three of them (JS25R, NAU-B3, B25) have a plasmid of similar size also containing a *rap* sequence (Table 4). The *rap* genes encoded by the plasmids of JS25R (CP009680, 8438 bp) and NAU-B3 (HG514500, 8439 bp) showed 99% identity with the *rap* gene of pS499 at nucleotide level. In contrast, the *rap* gene located on the plasmid of B25 (LN999830, 8138 bp) does not share homology with that located on pS499. However, the B25 Rap protein shows 47% identity with the chromosome-encoded RapI of S499 at amino acid level.

**Table 4:** **Plasmids similar to pS499 present in ***Bacillus amyloliquefaciens*** and ***Bacillus subtilis*** strains**.

**Strain**	**Plasmid name**	**Plasmid (accession number)**	***Rap* gene (locus tag)**	**Identity (%)**
*B. amyloliquefaciens* subsp. *plantarum* JS25R	pBMJS25R	CP009680	NG74_RS19355	99
*B. amyloliquefaciens* subsp. *plantarum* NAU-B3	pBAMMD1	HG514500	BAPNAU_RS20545	99
*B. amyloliquefaciens* subsp. *plantarum* B25	II	LN999830	BAMMD1_RS18490	–
*B. amyloliquefaciens* subsp. *amyloliquefaciens* LL3	pMC1	CP002635	LL3_RS20265	–
*B. subtilis* IAM 1232	pTA1040	NC_001764	pTA1040_p6	65
*B. subtilis* IFO 3022	pTA1060	NC_001766	pTA1060_p7	71

*The plasmids harboring a rap gene are reported in the table (sequence similarity with the pS499 gene is expressed as the percentage of nucleotide identity)*.

The very low occurrence of a plasmid similar to pS499 also extends to the taxon *B. amyloliquefaciens* subsp. *amyloliquefaciens*, where only 2 out of 36 strains possess a plasmid. Specifically, the plasmid of strain MBE1283 (CP013728, 13,003 bp) has no *rap* genes, while the plasmid of strain LL3 (CP002635, 6758 bp) includes a *rap* gene that is not homologous to the one encoded by pS499 (Table 4) even if it shares 38% identity with the S499 chromosome-encoded RapI at amino acid level.

In *B. subtilis*, out of 11 small plasmids (ranging from 2246 to 8737 bp), only two (pTA1040, 7837 bp, and pTA1060, 8737 bp) include *rap* sequences (*rap40* and *rap60*) displaying respectively 65 and 71% identity with the *rap* gene of pS499 at nucleotide level (Table 4). Other *rap* genes encoded by bigger plasmids (75–85 Kb) do not show identity with the *rap* gene of pS499.

In addition, we were also able to rule out the presence of analogous plasmids in six other *Bacillus* spp. strains from our lab collection (23, 76, 98R, 98S, 104, GA1), since no bands were observed in gel electrophoresis after plasmid DNA extraction and no pS499 *rap* gene was amplified by PCR (data not shown).

### The presence of plasmid pS499 affects growth kinetics and proteolytic activity

An S499 colony that had lost the plasmid was isolated after 14 culture cycles in sub-inhibitory conditions. On LBA, the colonies of the cured strain, named S499 P^−^, were morphologically different and more precisely, smoother, and larger compared to colonies of the wild-type S499. The 16S rDNA and the sequences of *gyrA* and *cheA* genes of S499 P^−^ were amplified, sequenced, and aligned to the respective sequences of S499 to prove that S499 P^−^ was a derivative of S499. For each gene, the PCR products shared 100% nucleotide sequence identity (Supplementary Data Sheet).

**Figure 4:**
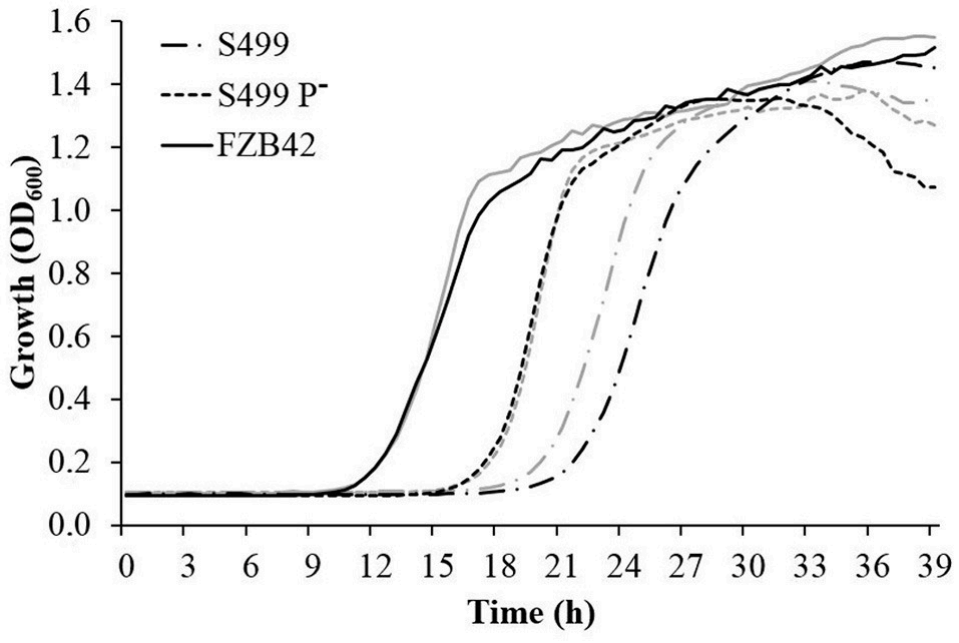
**Effects of plasmid curing and medium composition on bacterial growth**. *Bacillus amyloliquefaciens* subsp. *plantarum* FZB42, S499 and its plasmid-cured derivative, S499 P^−^, were grown in LB broth (black lines) and modified LB broth (gray lines) at 28°C. The assays were carried out in 48-well plates, and optical density at 600 nm (OD_600_) was read every 30 min. Reported OD_600_ values correspond to the averages of reads from three different wells in one representative experiment.

**Figure 5:**
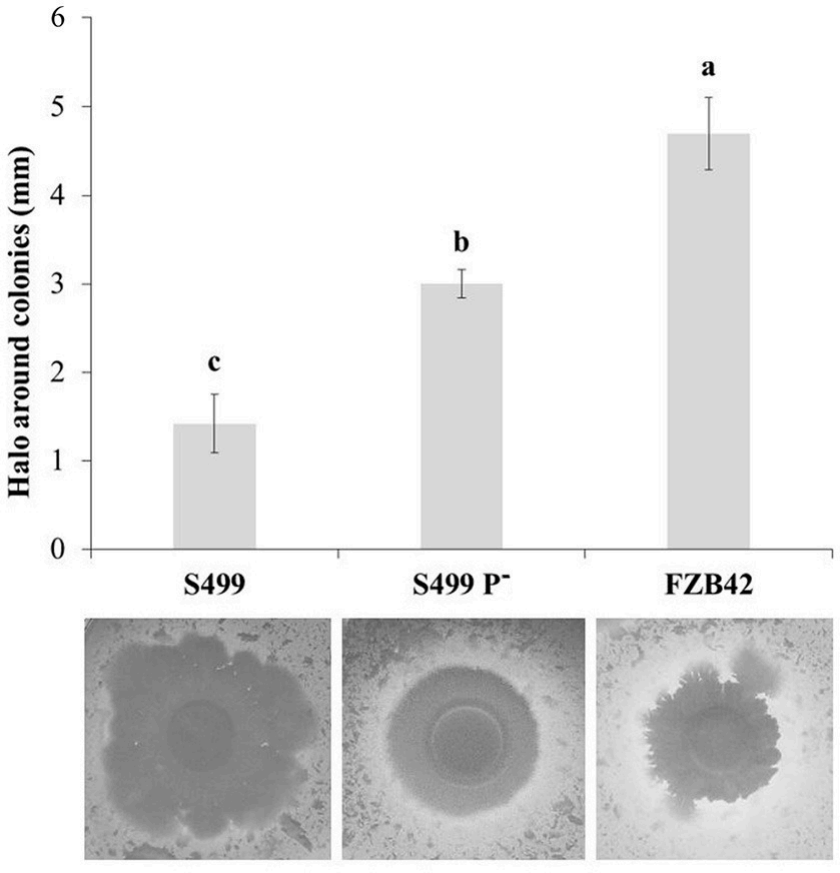
**Extracellular proteolytic activity**. The size of the clarification halos around the colonies of *Bacillus amyloliquefaciens* subsp. *plantarum* FZB42, S499 and its plasmid-cured derivative, S499 P^−^ was measured on LBA amended with 1% (w/v) skimmed milk plates at 48 h post inoculation. Results correspond to the average values of 10 replicates. Error bars represent standard errors. Different letters indicate significant differences according to Tukey's test (α = 0.05).

Following cultivation in LB broth, the growth dynamics of S499 P^−^ clearly differed from the parental strain and were actually more similar to the kinetics observed for FZB42, with a reduced lag phase and an increased growth rate. The logarithmic phase lasted 5.3 ± 0.2 h (average ± standard error) for S499 P^−^, while for S499 and FZB42 it lasted 6.3 ± 0.3 h and 6.3 ± 0.2 h respectively. However, S499 P^−^ had a lower final OD_600_ compared to S499 and FZB42 (Figure 4, black lines). Given these trends, we tested whether the faster cellular growth of S499 P^−^ could be linked to higher proteolytic activity, liberating amino acids as substrates from the proteinaceous source. First, we observed that substitution of tryptone by casamino acids in LB broth did not affect the cell growth of FZB42 and S499 P^−^, whereas it led to faster entry in the logarithmic phase in S499 (Figure 4, gray lines). The hypothesis of higher proteolytic activity of S499 P^−^ compared to S449 was then confirmed by testing the size of clarification halos forming around the colonies on LBA amended with skimmed milk. Indeed, after 48 h of incubation, the clarification halo around S499 P^−^ (3.0 ± 0.2 mm; average ± standard error) was larger than that of S499 (1.4 ± 0.3 mm), but smaller than the halo around FZB42 colonies (4.7 ± 0.4 mm) (Figure 5). These results were also supported by the fact that, after 6 h growth in LB medium, the release of extracellular proteases was greater in S499 P^−^ than in the wild type, determining slightly higher digestion of azocasein (Figure S1).

### Plasmid pS499 influences lipopeptide production

UPLC-ESI-MS analysis of culture filtrates at the end of the bacterial growth revealed that S499 P^−^ produced more surfactins (120.35 ± 8.29 μg ml^−1^, average ± standard error) and fengycins (39.60 ± 7.41 μg ml^−1^), but fewer iturins (32.30 ± 10.08 μg ml^−1^) than S499 (95.54 ± 1.33 μg ml^−1^, 22.29 ± 7.02 μg ml^−1^ and 56.65 ± 13.99 μg ml^−1^, respectively), although not significantly. FZB42 instead produced many more fengycins (238.10 ± 14.43 μg ml^−1^) and iturins (211.05 ± 24.01 μg ml^−1^), but fewer surfactins (84.10 ± 7.05 μg ml^−1^) than S499 and S499 P^−^ (Figure S2).

**Figure 6:**
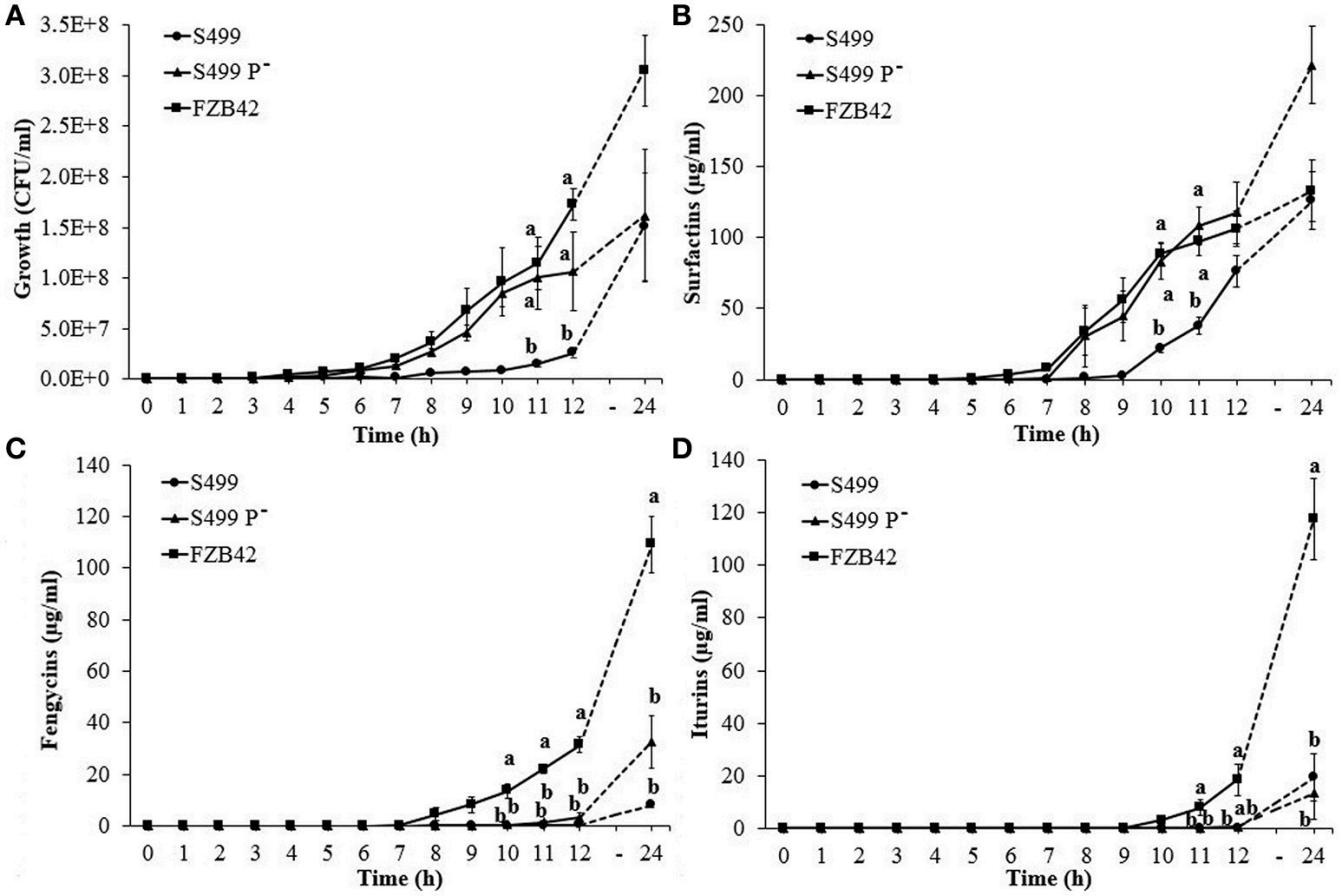
**Kinetics of cyclic lipopeptide production**. Growth curves **(A)** and surfactin **(B)**, fengycin **(C)**, and iturin **(D)** production in *Bacillus amyloliquefaciens* subsp. *plantarum* FZB42, S499 and its plasmid-cured derivative, S499 P^−^. Values correspond to the resulting averages of standardized data [Z = (X/μ)^*^100] from three independent experiments. Error bars represent standard errors and different letters indicate significant differences according to Tukey's test (α = 0.05).

To better understand the role of the plasmid in CLP production, we set up a new growth assay that allowed us to follow the kinetics of production during culture. In line with the OD_600_ results, CFU counts indicated that S499 P^−^ grew faster than S499, showing significant differences compared to the growth curve of S499 at 11 and 12 h, but not differently from FZB42 in these conditions (Figure 6A). The production of surfactins by S499 P^−^, similarly to FZB42, started earlier compared to S499, resulting in a significant increase in surfactin concentrations measured after 10 and 11 h of incubation. At 12 h, S499 P^−^ and FZB42 had released 118 ± 21 μg ml^−1^ (average ± standard error) and 106 ± 12 μg ml^−1^ of surfactins respectively, while S499 produced only 76 ± 11 μg ml^−1^. At the final time-point (24 h), the trend for surfactin production by S499 P^−^ (222 ± 27 μg ml^−1^) was also higher than for FZB42 (133 ± 22 μg ml^−1^), although not significantly (Figure 6B).

Surfactins were detected in the culture filtrates of the three strains 3 h after *srfA* gene expression started to increase. Regression analysis of surfactin production and *srfA* gene expression levels assessed 3 h before was highly significant (*p* < 0.01, *y* = 1.7059x + 15.624, *R*^2^ = 0.283). In accordance with surfactin production rates, *srfA* gene expression in S499 P^−^ and FZB42 increased earlier than in S499. Specifically, transcription levels of the *srfA* gene increased after 5 h of incubation and production started after 8 h in FZB42 and S499 P^−^, whereas in S499 gene expression increased after 7 h and surfactins were detected after 10 h (Figure S3). When the expression of the plasmid-encoded *rap* gene was assessed in S499, its transcripts were detected at similar level of *gyrA* gene expression during early (ΔC_T_ = −0.69 ± 0.66; average ± standard error), middle (ΔC_T_ = 0.61 ± 0.65) and late growth phases (ΔC_T_ = 1.80 ± 1.12).

Kinetic tests showed no significant differences in the production of fengycins (detected from 10 h after incubation) in S499 (0.04 ± 0.03 μg ml^−1^, average ± standard error) and S499 P^−^ (0.55 ± 0.18 μg ml^−1^), and production was significantly lower than in FZB42 (13.53 ± 2.70 μg ml^−1^) (Figure 6C). Similar behavior was observed for iturins, which were only detected in the culture filtrates of S499 and S499 P^−^ starting from 12 h after incubation, and in lower quantities (0.10 ± 0.08 and 0.70 ± 0.43 μg ml^−1^) compared to the amounts detected in FZB42 supernatants (18.49 ± 5.85 μg ml^−1^) (Figure 6D). However, at the end of the experiment (24 h) we observed an increasing although not significant trend for the production of fengycins and lower production of iturins by S499 P^−^ compared to S499, in accordance with previous assays.

### The impact of plasmid pS499 on motility and biofilm formation

**Figure 7:**
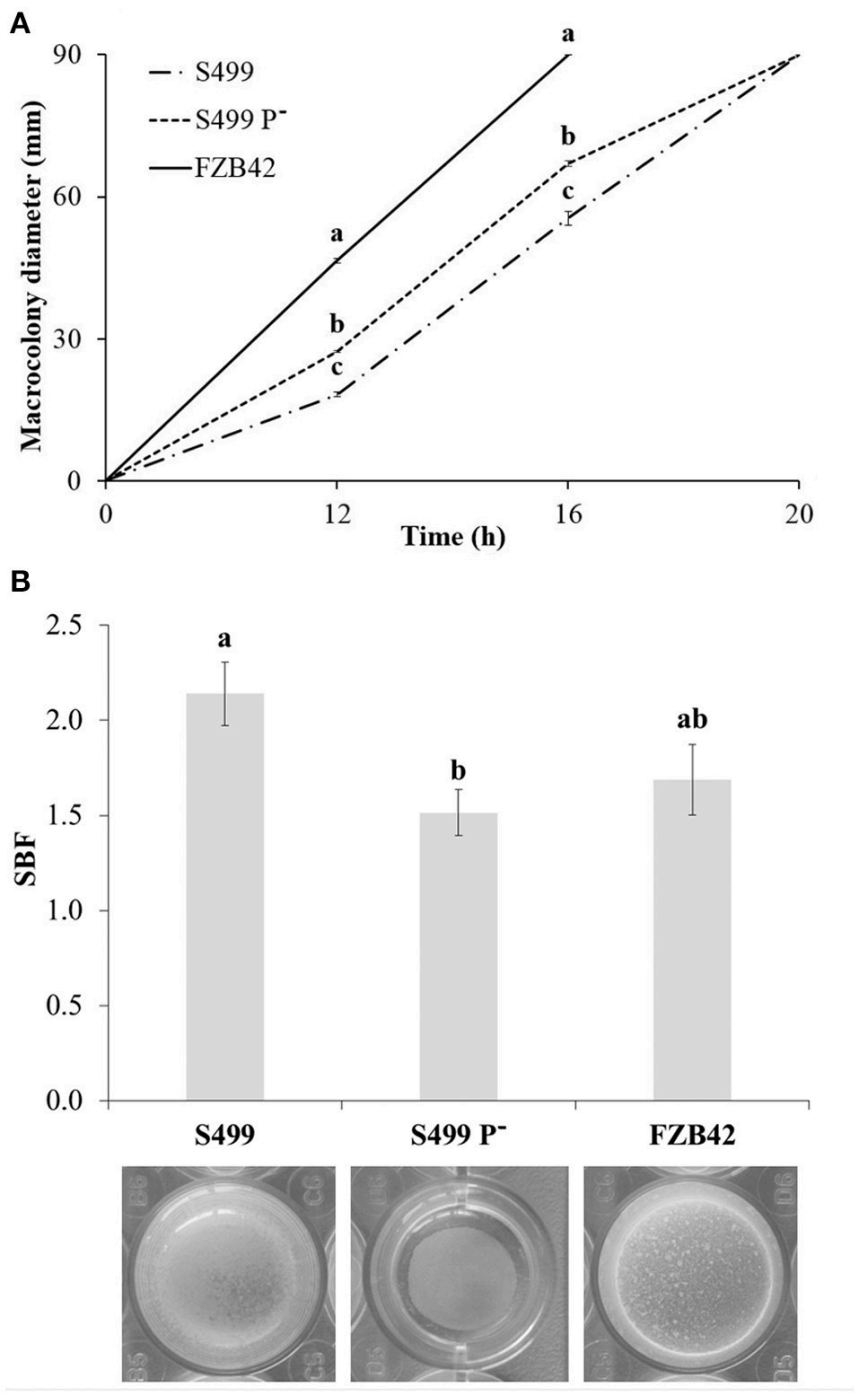
**Impact on swarming motility and biofilm formation**. Values representing swarming motility on LBA (agar 0.5%) **(A)** correspond to the averages of macrocolony diameters measured in dishes from one representative experiment (four replicates). Error bars represent standard errors. Different letters indicate significant differences according to Tukey's test (α = 0.05). Specific biofilm formation (SBF) was measured after 72 h of incubation at 27°C in LB Broth **(B)**. Values correspond to the averages of eight replicates in one representative experiment. Error bars represent standard errors. Different letters indicate significant differences according to Tukey's test (α = 0.05).

Given the key role of surfactin in motility and biofilm formation in *Bacillus* (Raaijmakers et al., 2010), earlier and more abundant release of this CLP by S499 P^−^ could have an impact on these two phenotypic traits. Indeed, after 12 h of incubation, the diameter of S499 P^−^ macrocolonies (27.3 ± 0.2 mm, average ± standard error) was significantly larger than that observed for the parental strain (18.3 ± 0.4). The difference between S499 P^−^ and S499 was retained during the experiment, with their colonies measuring 67.0 ± 0.6 mm and 55.5 ± 1.5 mm respectively after 16 h. On the other hand, FZB42 was significantly faster than both S499 P^−^ and S499. Indeed, its average macrocolony diameter was 46.5 ± 0.4 mm after 12 h of incubation, and the colonies already reached the edge of the dishes after 16 h. After 20 h of incubation, the surface of each dish was fully covered by the bacterial cells for all strains (Figure 7A).

Conversely, the ability to form biofilm was reduced in S499 P^−^ compared to both S499 and FZB42. Indeed, after 72 h of static incubation, the SBF values of S499 P^−^ (1.51 ± 0.12, average ± standard error) were significantly lower compared to S499 (2.14 ± 0.17) and FZB42 (1.69 ± 0.19). S499 P^−^ produced a pellicle at the liquid-air interface that was not attached to the edge of the wells, as it was for S499 and FZB42 (Figure 7B).

### Plasmid pS499 influences fengycin and iturin-dependent antifungal activity

**Figure 8:**
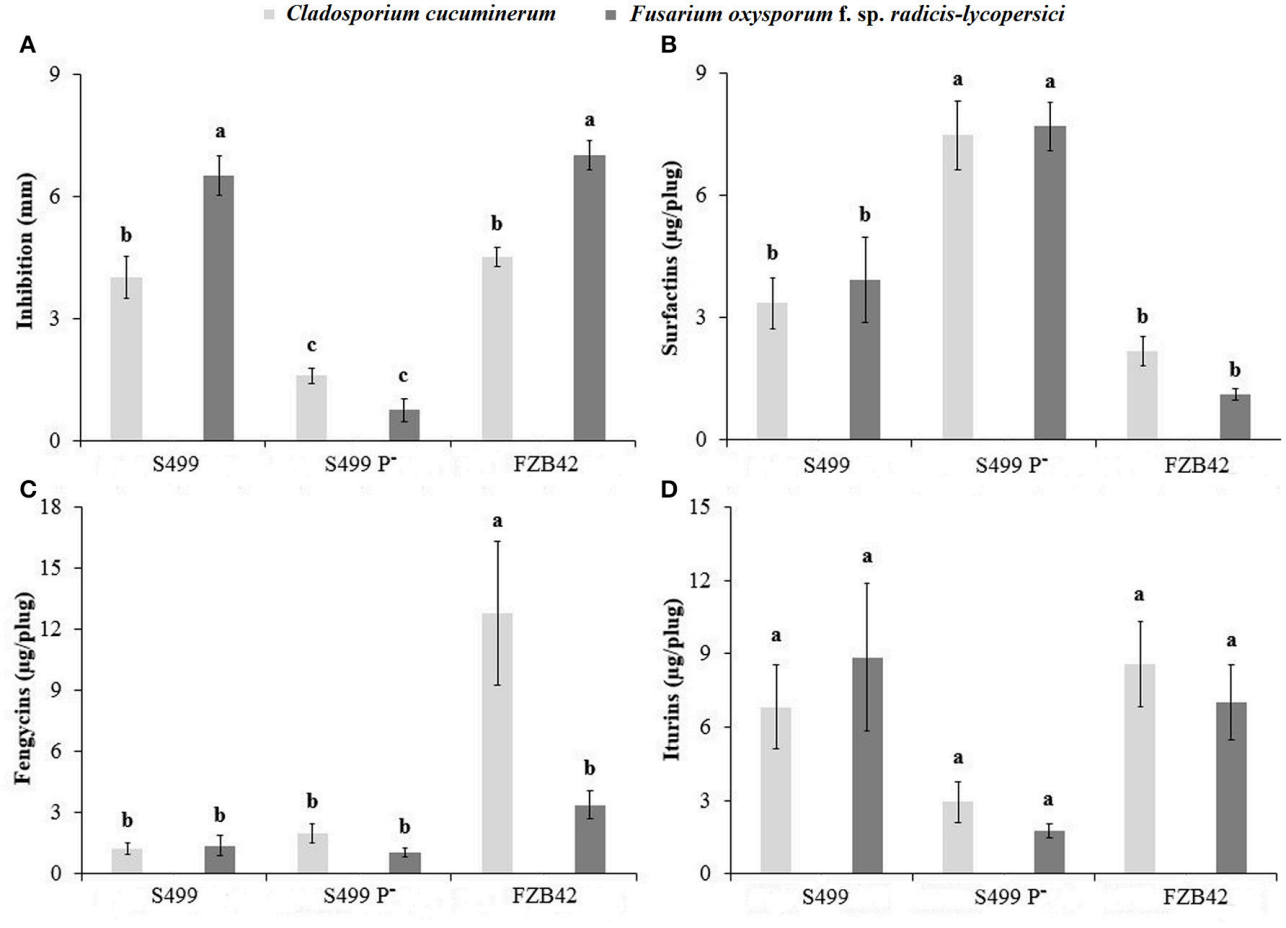
**Antifungal activity**. Inhibition zone **(A)** and concentration of surfactins **(B)**, fengycins **(C)**, and iturins **(D)** produced by *Bacillus amyloliquefaciens* subsp. *plantarum* FZB42, S499 and its plasmid-cured derivative, S499 P^−^, measured after 72 h of incubation in the presence of the pathogen in LBA dishes. Light gray and dark gray histograms refer to *Cladosporium cucuminerum* and *Fusarium oxysporum* f. sp. *radicis-lycopersici* respectively. An *F*-test revealed non-significant differences between experiments and data were pooled. Means and standard error values were calculated as the pool of six replicates (Petri dishes) from two experiments. Different letters indicate significant differences according to Tukey's test (α = 0.05).

Since S499 produces fengycins and iturins, which show direct antifungal activity, we further investigated the effect of pS499 on fungal growth inhibition potential *in vitro*. Multifactorial ANOVA indicated a significant effect of the pathogen type (*p* < 0.01), the *Bacillus* strain (*p* < 0.01) and the pathogen × *Bacillus* strain (*p* < 0.01) on the inhibition zones. The S499 P^−^ strain was less effective than S499 and FZB42 in inhibiting the growth of *C. cucumerinum* and *F. oxysporum* f. sp. *radicis-lycopersici*, S499 and FZB42 were significantly more effective against *F. oxysporum* f. sp. *radicis-lycopersici* than against *C. cucumerinum*. Indeed, the inhibition zone was smaller for S499 P^−^ (1.6 ± 0.2 mm against *C. cucumerinum* and 0.7 ± 0.3 mm against *F. oxysporum* f. sp. *radicis-lycopersici*) compared to S499 (4.0 ± 0.5 mm/6.5 ± 0.5 mm) and FZB42 (4.5 ± 0.2 mm/7.0 ± 0.4 mm) (Figure 8A).

In these confrontation assays performed on gelified LB medium, bacterial cells evolve as microcolonies that can be viewed as a kind of biofilm-structured non-motile community. Their physiology may thus be quite different compared to the planktonic state, with a possible impact on CLP synthesis. Plugs of medium were removed from the inhibition zone to quantify the CLP pattern through UPLC-ESI-MS analysis. Regression analysis indicated a significant effect of iturin (positive, *p* < 0.01, *y* = 0.256x + 2.522, *R*^2^ = 0.245) and surfactin concentration (negative, *p* < 0.01, *y* = −0.618x + 6.710, *R*^2^ = 0.541) on the size of inhibition zone, but not for fengycins.

Multifactorial ANOVA indicated a significant effect of the *Bacillus* strain (*p* < 0.01) on surfactin production, of the pathogen type (*p* < 0.01), the *Bacillus* strain (*p* < 0.01), and the pathogen × *Bacillus* strain (*p* < 0.01) on fengycin production, and of the *Bacillus* strain (*p* < 0.01) on iturin production. Surfactin production by S499 P^−^ (7.47 ± 0.84 μg plug^−1^ from the inhibition zone of *C. cucumerinum* and 7.69 ± 0.75 μg plug^−1^ from the inhibition zone of *F. oxysporum* f. sp. *radicis-lycopersici*) was significantly higher than by S499 (3.36 ± 0.63 μg plug^−1^ and 3.94 ± 1.05 μg plug^−1^ respectively) and FZB42 (2.18 ± 0.35 μg plug^−1^ and 1.12 ± 0.14 μg plug^−1^ respectively) (Figure 8B). Fengycin production seemed to be stimulated by the presence of *C. cucumerinum*, but only in the case of FZB42. Indeed, in FZB42—*C. cucumerinum* dual cultures we detected 12.80 ± 3.50 μg plug^−1^ of fengycins, whereas in FZB42—*F. oxysporum* f. sp. *radicis-lycopersici* dual cultures we detected 3.37 ± 0.67 μg plug^−1^ of fengycins. The quantity of fengycins extracted from the inhibition zones around the colonies of S499 P^−^ and S499 was similar and comprised between 1.03 ± 0.23 and 1.97 ± 0.46 μg plug^−1^ (Figure 8C). Iturin production was lower in S499 P^−^ compared to S499, in line with the trend observed in liquid cultures. Indeed, we detected only 2.93 ± 0.84 μg plug^−1^ of iturins in S499 P^−^— *C. cucumerinum* dual cultures and 1.76 ± 0.30 μg plug^−1^ in S499 P^−^—*F. oxysporum* f. sp. *radicis-lycopersici* dual cultures, while we extracted 6.83 ± 1.71 μg plug^−1^ and 8.85 ± 3.01 μg plug^−1^ from the respective inhibition zones of S499 (Figure 8D).

## Discussion

The efficacy of some *B. amyloliquefaciens* subsp. *plantarum* strains available on the market as biofungicides (Borriss, 2011) can be improved after elucidating the molecular mechanisms that govern the multiple interactions occurring among beneficial bacteria, pathogenic microorganisms, host plants and the environment (Raaijmakers et al., 2009; Dutta and Podile, 2010; Berendsen et al., 2012). Convinced that the availability of the bacterial genomes may foster this elucidation, we compared the genomes of S499 and the well-characterized FZB42 (Chen et al., 2007) to find unique characteristics that may explain the S499 distinctive phenotype (Ongena et al., 2005b; Cawoy et al., 2014, 2015).

The genome comparison showed a high degree of genetic conservation between the two strains. A large percentage of sequence identity was found for the genes responsible for root colonization, plant growth promotion, and biocontrol. Therefore, we hypothesize that divergences in regulatory elements could explain the different behavior of the bacteria, especially influencing their sensing of and adaptation to the environment. Indeed, some specific genes were identified as transcriptional factors or annotated as hypothetical proteins and it would be challenging to analyse how they contribute to determining the phenotypic differences observed between S499 and FZB42. Some other unique CDS encode transport proteins, such as ABC transporter permeases (e.g., AS588_RS00475, AS588_RS09125, RBAM_RS18310) and other transmembrane proteins (e.g., RBAM_RS01165, RBAM_RS02920), that might play a role in perceiving and responding to the environment in different ways for S499 and FZB42. In general, cell membrane transport systems are crucial for the survival of many microorganisms in natural conditions (Konings, 2006). For example, surface colonization relies on the efficient transfer of potassium ions (K^+^) in *B. subtilis*, since a mutation in the K^+^ transporter KtrAB prevents cell spreading (Kinsinger et al., 2005).

Within unique CDS in the S499 genome, phage-related sequences were also more numerous. Phages represent one of the main channels in horizontal gene transfer, therefore genetic variability among bacterial strains is significantly dependant on prophage acquisition (Brüssow and Hendrix, 2002). According to Wang et al. (2010), cryptic prophages help the host to overcome adverse environmental conditions (oxidative, osmotic, and acid stress). An abundance of prophage regions could reflect a higher plasticity of the S499 genome. This hypothesis is also supported by the presence of genomic rearrangements and plasmid DNA, which are other features contributing to genetic variation in bacteria and their rapid evolution (Van Elsas et al., 2000; Dobrindt and Hacker, 2001).

The presence of a plasmid (pS499) emerged as a distinctive feature of S499, as shown by our comparative analysis. This is an interesting aspect, given the fact that plasmids are not frequent either in the available sequenced strains or in other *Bacillus* spp. strains (23, 76, 98R, 98S, 104, GA1) also identified as good antibiotic producers in our laboratory (Cawoy et al., 2015). Based on its size and structural organization, pS499 can be ascribed to the class of small rolling circle replicons (Guglielmetti et al., 2007). These natural plasmids have a typical organization that includes a replication module, a gene responsible for plasmid mobilization and one or more modules not involved in plasmid metabolism but encoding traits related to the host physiology. For instance, in some strains the plasmid encodes a heat shock protein putatively involved in stress responses, which may represent an advantage for the bacterium in its natural environment (Thorsted et al., 1999). Some impact of pS499 on the physiology of S499 can be expected from the presence of a *rap-phr* cassette. Indeed, some plasmid-encoded response regulator aspartate phosphatases have already been proven to control extracellular protease production, biofilm architecture, sporulation, and the genetic competence of *B. subtilis* and *B. amyloliquefaciens* (Meijer et al., 1998; Koetje et al., 2003; Parashar et al., 2013; Boguslawski et al., 2015). As the *rap* gene located on pS499 was expressed in S499 cells from an early growth phase, a role in regulating the cellular physiology of the bacterium can be foreseen.

Chromosome-encoded Rap-Phr systems have been extensively studied in *B. subtilis*, where they control cellular processes regulated by two-component systems, such as competence development, antibiotic synthesis, protein secretion, and sporulation (Perego et al., 1994; Bongiorni et al., 2005; Auchtung et al., 2006). Rap proteins (11 members in *B. subtilis*) counteract kinases by dephosphorylating intracellular response regulators (e.g., Spo0F) or alternatively, they inhibit transcriptional factors such as ComA and DegU in their DNA binding activity (Bongiorni et al., 2005). Seven of the 11 characterized *rap* genes are followed by a *phr* gene, encoding a phosphatase regulator (Phr) precursor peptide. Phr peptides are extracellularly processed to pentapeptides. Once they reach a critical concentration, therefore acting as quorum sensing signals, the mature peptides are reimported through an oligopeptide permease (Opp) into the cytoplasm, where they inhibit Rap proteins (Pottathil and Lazazzera, 2003). Besides the *rap-phr* cassette located on pS499, we also identified 10 *rap* genes in the S499 chromosome, with *rapI_1* representing another difference between S499 and FZB42. Having *rapI_1* and an additional *rap-phr* cassette encoded by a plasmid, which can further increase its copy number, could constitute an ecological advantage for S499 compared to FZB42. Interestingly, Thorsted et al. (1999) highlighted that the presence of plasmid-encoded *rap* genes is more diffuse among Russian strains isolated from the soil rather than in strains selected to be used in the Japanese fermentation industry. By curing S499 of its plasmid, we aimed to elucidate the role played by the Rap-Phr system encoded by pS499 in the phenotypic differences that have so far been characterized in S499 and FZB42.

The absence of the *rap-phr* cassette located on pS499 caused faster growth of S499 P^−^ compared to the wild strain, which could be linked to improved substrate utilization ability by the cured cells. Indeed, increased activity of secreted proteases was observed for S499 P^−^. The increase in proteolytic activity is in accordance with previous studies showing that the pTA1060-encoded Rap60-Phr60 system controls the secretion of proteolytic enzymes. More precisely, *rap60* is involved in down-regulation of the *aprE* gene (responsible for production of the extracellular protease subtilisin) during post-exponential growth (Koetje et al., 2003; Boguslawski et al., 2015) through a complex molecular pathway. The *rap* gene of pS499 displays 71% sequence identity with *rap60* at nucleotide level. Nevertheless, in our case, increased proteolytic activity of S499 P^−^ was also observed during the early growth phase (6 h), with very low biomass. Thus, it is conceivable that the *rap-phr* cassette of pS499 may behave differently from pTA1060-encoded Rap60-Phr60, although the same negative control on the secretion of extracellular proteases can be assumed. Consistently with the earlier start of exponential growth phase for FZB42, both azocasein and skimmed milk assays showed that FZB42 extracellular proteolytic activity was higher compared to S499 P^−^. Therefore, in addition to the plasmid, other genetic features are responsible for the different phenotypes of FZB42 and S499.

The higher growth rate of S499 P^−^ can only partially be explained by the increased production of proteases. Indeed, in modified LB cultures the S499 curve did not overlap that of S499 P^−^, but still rose later and showed a milder slope. Another possible explanation for the observed growth shift is related to the energy cost of pS499 replication in S499 cells. Indeed, the presence of plasmids is frequently associated with reduced growth rates, especially in the case of large and high copy number plasmids (Smith and Bidochka, 1998; Diaz Ricci and Hernández, 2000). For example, Trautwein et al. (2016) recently reported that deletion of the 262 Kb native plasmid in *Phaeobacter inhibens* DSM 17395 improved the growth efficiency of the strain. They hypothesized that either the plasmid delays cell division by slowing down the DNA replication process, or that it has a metabolic weight that compromises growth efficiency. Even if pS499 is considerably reduced in size, we can assume a similar fitness cost for the host cells. However, it is worth noting that S499 tended to keep its plasmid during cell multiplication, despite the hypothesized energy cost. In fact, although exposed to sublethal conditions during our curing procedure, plasmid loss was a rare event in S499. This corroborates the hypothesis that pS499 could provide a real selective advantage in natural environments, as in the case of pQBR103 from *Pseudomonas fluorescens* for example (Lilley and Bailey, 1997) and many other bacterial plasmids (Thomas, 2004).

Plasmid curing considerably affected the CLP production by S499. Kinetic assay showed that S499 P^−^ behaves similarly to FZB42 as far as surfactin is concerned, suggesting a role of the plasmid in regulating surfactin synthesis. However, it remains unclear whether earlier production by S499 P^−^ is due to earlier entrance in the exponential phase or to divergences in transcriptional regulation. Indeed, it is known that *srfA* gene expression is cell-density dependent, being controlled by the ComP-ComA signal transduction system (Nakano et al., 1991). Given the differences observed in growth rates, earlier surfactin production could be related to faster multiplication of S499 P^−^ and FZB42 populations. Nevertheless, we noted that the cured strain accumulated many more surfactins than FZB42 and S499 over time. Consistently, *srfA* gene expression was down-regulated in *B. subtilis* OKB105 transformed with the plasmid-encoded *rap* gene from *B. amyloliquefaciens* subsp. *plantarum* NAU-B3 (99% nucleotide sequence identity with pS499 *rap*; Yang et al., 2015). Furthermore, it has been demonstrated that the Rap phosphatase modulates *srfA* transcription by forming a ternary complex with ComA and *srfA* promoter (Yang et al., 2015). Similarly, we can postulate that loss of the pS499 *rap* gene enhanced *srfA* expression because of the removal of inhibition in the cured strain. Conversely, no significant differences between the cured strain and its parental strain were observed in terms of the release of fengycins and iturins. We can therefore assume that the differences between S499 and FZB42 observed in the production of these CLPs mostly depend on a different regulatory pathway, not involving pS499.

As a consequence of different surfactin production, in S499 P^−^ some phenotypic traits related to rhizosphere competence were affected, e.g., the speed of surface colonization. *Bacillus* spp. are capable of multicellular behavior known as swarming motility, a common bacterial way of moving across surfaces powered by rotating flagella (Kearns and Losick, 2003). Swarming motility is highly dependent on the secretion of surfactins, which reduce surface tension given their amphiphilic nature (Kinsinger et al., 2003; Leclère et al., 2006; Kearns, 2010). Therefore, a boost in surfactin production by S499 P^−^ can explain the increase in its swarming ability. Similarly, the faster surface spreading of FZB42 can be related to higher production of surfactins compared to S499, but not to S499 P^−^. Indeed, according to our kinetic assays, the surfactin production rates of S499 P^−^ and FZB42 were similar. Here we can speculate that the phenotypes were influenced by the different growth rates of the strains. Even without reaching a final conclusion on the mechanism involved, we can assume that the presence of the plasmid could be necessary in the process of root surface colonization by S499, because swarming motility is a major factor favoring root colonization, even more than chemotaxis (Gao et al., 2016).

*Bacillus* spp. develop in the rhizosphere in the form of biofilms, which are also crucial for the biocontrol of plant pathogens (Bais et al., 2004; Chen et al., 2012, 2013). SBF values were lower for FZB42 compared to S499 and on curing S499 we observed a further reduction in SBF in S499 P^−^. These results suggest a role for the plasmid in determining the difference found between S499 and FZB42. Surfactins function as paracrine signals that induce the differentiation of biofilm matrix producer cells (López et al., 2009). By regulating the phosphorylation state of DegU, ComA, and Spo0A, different Rap phosphatases control multiple signaling cascades. This redundant network can integrate exogenous and endogenous signals, leading to the formation of distinct biofilm subpopulations (motile cells, matrix producers, competent cells, cannibals, etc.; Mielich-Süss and Lopez, 2015). In the light of the increased and earlier surfactin production associated with plasmid curing, we would have expected an increase rather than a reduction in SBF. However, it is likely that very small amounts of this lipopeptide are sufficient to trigger biofilm formation and that other factors become more important later. It has indeed been observed that the surfactin-producing subpopulation is actually restrained in biofilm layers (Mielich-Süss and Lopez, 2015). Although considerable effort is required to understand which specific pathway is targeted by the pS499-encoded Rap, we can assume that its lack is most probably the cause of the altered phenotype observed for S499 P^−^. Likewise, McLoon et al. (2011) showed that RapP encoded by an 80 Kb plasmid from *B. subtilis* NCIB 3610 is required for the formation of robust biofilms typical of wild-type strains. Later studies revealed that RapP is a Spo0F phosphatase and that it is involved in the phosphorelay modulating the expression of *epsA-O* and *yqxM-sipW-tasA* biofilm operons (Parashar et al., 2013).

Similarly to previous results (Cawoy et al., 2015), regression analysis of the antagonism assay showed that limited iturin production affected the intrinsic ability of S499 P^−^ to inhibit the mycelial growth of *C. cucumerinum* and *F. oxysporum* f. sp. *lycopersici*. Furthermore, it is conceivable that the negative correlation with surfactin concentration resulted from a physiological imbalance: the more resources were allocated to the synthesis of surfactins, in particular by S499 P^−^, the fewer were available for synthesis of iturins, which are known to be produced in the stationary phase of bacterial growth (Jacques et al., 1999). Plasmid pS499 is therefore indirectly relevant for the antifungal activity of the strain, being involved in the modulation of lipopeptide production. At all events, the fact that S499 and FZB42 produced similar quantities of iturins in dual culture with the pathogens suggests that other genetic traits are also involved.

In conclusion, our data show that the plasmid-encoded *rap* gene of *B. amyloliquefaciens* subsp. *plantarum* S499 has a role in controlling several traits like protease secretion, production of surfactins and biofilm formation. Growth and motility are also influenced, either indirectly by the pS499 Rap-Phr system and/or by the presence of the plasmid itself. To our knowledge, we provide here the first report on the relationship between a plasmid, or control of Rap phosphatase on fengycin and iturin production, and the related impact on biocontrol. To illustrate these molecular pathways more exhaustively, further studies on the cellular mechanisms are necessary. Finally, by comparing the behavior of FZB42 and the S499 plasmid-cured derivative S499 P^−^, we can conclude that pS499 plays a significant role in the phenotype of the two strains, although other genetic differences merit additional investigation.

## Author contributions

GM carried out all the experiments, analyzed the data and wrote and edited the manuscript. LF carried out UPLC-ESI-MS analysis and wrote and edited the manuscript. SS carried out qRT-PCR and wrote and edited the manuscript. GP, IP, and MO conceived the work, designed the experiments, analyzed the data and wrote and edited the manuscript. All the authors have read the manuscript and agreed to its content.

### Conflict of interest statement

The authors declare that the research was conducted in the absence of any commercial or financial relationships that could be construed as a potential conflict of interest.
