# Supplementary material for: Key Impact of an Uncommon Plasmid on Bacillus amyloliquefaciens subsp. plantarum S499 Developmental Traits and Lipopeptide Production
Source: Front Microbiol. 2017 Jan 19;8:17. doi: 10.3389/fmicb.2017.00017 (PMC5243856; doi:10.3389/fmicb.2017.00017)
Supplement: Supplementary file 1 [file Table1.DOCX]

**Table S1. Microorganisms used in this study.**

| **Phylum** | **Strain** | **ID** |
| --- | --- | --- |
| Bacteria | *Bacillus amyloliquefaciens* subsp. *plantarum* S499 | S499 |
|  | *Bacillus amyloliquefaciens* subsp. *plantarum* S499 P^-^ | S499 P^-^ |
|  | *Bacillus amyloliquefaciens* subsp. *plantarum* FZB42 | FZB42 |
|  | *Bacillus amyloliquefaciens* GA1 | GA1 |
|  | *Bacillus subtilis/amyloliquefaciens* 23 | 23 |
|  | *Bacillus subtilis/amyloliquefaciens* 76 | 76 |
|  | *Bacillus subtilis/amyloliquefaciens* 98R | 98R |
|  | *Bacillus subtilis/amyloliquefaciens* 98S | 98S |
|  | *Bacillus subtilis/amyloliquefaciens* 104 | 104 |
| Fungi | *Cladosporium cucumerinum* |  |
|  | *Fusarium oxysporum* f. sp. *radicis-lycopersici* |  |
