# Supplementary material for: Key Impact of an Uncommon Plasmid on Bacillus amyloliquefaciens subsp. plantarum S499 Developmental Traits and Lipopeptide Production
Source: Front Microbiol. 2017 Jan 19;8:17. doi: 10.3389/fmicb.2017.00017 (PMC5243856; doi:10.3389/fmicb.2017.00017)
Supplement: Supplementary file 2 [file Table2.DOCX]

**Table S2. Genetic sequences analysed to describe the frequency of plasmids similar to pS499.** Sequences were present on the NCBI Genome Assembly and Annotation reports for *Bacillus amyloliquefaciens* subsp. *plantarum* and *B. amyloliquefaciens* subsp. *amyloliquefaciens,* and the Plasmid Annotation report for *B. subtilis* (http://www.ncbi.nlm.nih.gov).

| **Species** | **Strain / Plasmid** | **Accession no.** |
| --- | --- | --- |
| *Bacillus amyloliquefaciens* subsp. *plantarum* | FZB42 | CP000560 |
|  | CAU B946 | HE617159 |
|  | YAU B9601-Y2 | HE774679 |
|  | M27 | AMPK00000000 |
|  | AS43.3 | CP003838 |
|  | UCMB5036 | HF563562 |
|  | UCMB-5033 | HG328253 |
|  | UCMB5113 | HG328254 |
|  | NAU-B3 | HG514499 |
|  | SK19.001 | AOFO00000000 |
|  | TrigoCor1448 | CP007244 |
|  | AH159-1 | JFBZ00000000 |
|  | SQR9 | CP006890 |
|  | W2 | JOKF00000000 |
|  | JS25R | CP009679 |
|  | AP183 | JXAM00000000 |
|  | GR4-5 | JYGH00000000 |
|  | KACC 13105 | JTKJ00000000 |
|  | NJN-6 | CP007165 |
|  | JJ-D34 | CP011346 |
|  | YJ11-1-4 | CP011347 |
|  | G341 | CP011686 |
|  | OB9 | LGAU00000000 |
|  | B26 | LGAT00000000 |
|  | KCTC 13012 | LHCC00000000 |
|  | NBIF-003 | LJJY00000000 |
|  | NRRL B-4257 | LLZC00000000 |
|  | KACC 18228 | LLZA00000000 |
|  | NRRL B-4257 | LLZB00000000 |
|  | FKM10 | LNTG00000000 |
|  | RC218 | LQCL00000000 |
|  | B25 | LN999829 |
| *Bacillus amyloliquefaciens* subsp. *amyloliquefaciens* | DSM7 | FN597644 |
|  | TA208 | CP002627 |
|  | LL3 | CP002634 |
|  | XH7 | CP002927 |
|  | Y2 | CP003332 |
|  | DC-12 | AMQI00000000 |
|  | EGD-AQ14 | AVQH00000000 |
|  | UASWS BA1 | AWQY00000000 |
|  | CC178 | CP006845 |
|  | LFB112 | CP006952 |
|  | EBL11 | JCOC00000000 |
|  | B1895 | JMEG00000000 |
|  | CMW1 | BBLH00000000 |
|  | X1 | JQNZ00000000 |
|  | HB-26 | AUWK00000000 |
|  | JJC33M | JTJG00000000 |
|  | LPL-K103 | JXAT00000000 |
|  | TF28 | JUDU00000000 |
|  | L-H15 | CP010556 |
|  | KHG19 | CP007242 |
|  | 12B | JZDI00000000 |
|  | L-S60 | CP011278 |
|  | 516_BAMY | JVEA00000000 |
|  | Lx-11 | AUNG00000000 |
|  | 629 | LGYP00000000 |
|  | Bs006 | LJAU00000000 |
|  | XK-4-1 | LJDI00000000 |
|  | RHNK22 | LMAG00000000 |
|  | Jxnuwx-1 | LMAT00000000 |
|  | MBE1283 | CP013727 |
|  | 11B91 | LPUP00000000 |
|  | B4140 | LQYO00000000 |
|  | B425 | LQYP00000000 |
|  | UMA56639 | CP006058 |
|  | UMAF6614 | CP006960 |
|  | B15 | CP014783 |
| *Bacillus subtilis* | pIM13 | [M13761](http://www.ncbi.nlm.nih.gov/nuccore/M13761) |
|  | pTA1040 | [U32378](http://www.ncbi.nlm.nih.gov/nuccore/U32378) |
|  | pTA1015 | [U32379](http://www.ncbi.nlm.nih.gov/nuccore/U32379) |
|  | pTA1060 | [U32380](http://www.ncbi.nlm.nih.gov/nuccore/U32380) |
|  | p1414 | [AF091592](http://www.ncbi.nlm.nih.gov/nuccore/AF091592) |
|  | pBS608 | [AY836798](http://www.ncbi.nlm.nih.gov/nuccore/AY836798) |
|  | pLS30 | [AB243053](http://www.ncbi.nlm.nih.gov/nuccore/AB243053) |
|  | pPL1 | [DQ140187](http://www.ncbi.nlm.nih.gov/nuccore/DQ140187) |
|  | pLS20 | [AB615352](http://www.ncbi.nlm.nih.gov/nuccore/AB615352) |
|  | pLS32 | [AB615353](http://www.ncbi.nlm.nih.gov/nuccore/AB615353) |
|  | pBS32 | [KF365913](http://www.ncbi.nlm.nih.gov/nuccore/KF365913) |
|  | pSU01 | [ANIP01000001](http://www.ncbi.nlm.nih.gov/nuccore/ANIP01000001) |
|  | unnamed1 | [CP014472](http://www.ncbi.nlm.nih.gov/nuccore/CP014472) |
|  | unnamed2 | [CP014473](http://www.ncbi.nlm.nih.gov/nuccore/CP014473) |
|  | pBEST195S | [AP011542](http://www.ncbi.nlm.nih.gov/nuccore/AP011542) |
