# Supplementary material for: Key Impact of an Uncommon Plasmid on Bacillus amyloliquefaciens subsp. plantarum S499 Developmental Traits and Lipopeptide Production
Source: Front Microbiol. 2017 Jan 19;8:17. doi: 10.3389/fmicb.2017.00017 (PMC5243856; doi:10.3389/fmicb.2017.00017)
Supplement: Supplementary file 3 [file Table3.docx]

**Supplementary Table 3. Primers used in this study.** Oligonucleotides were designed by Primer3web version 4.0.0 (Untergasser et al., 2012).

| **Target gene** | **Primers** | **Sequence (5’-3’)** | **References** |
| --- | --- | --- | --- |
| *16S rRNA* | 9-F | GAGTTTGATCCTGGCTCAG | Weisburg et al. (1991) |
|  | 1512-R | ACGGCTACCTTGTTACGACTT |  |
| *cheA* | CheA-F | AGAGCTGCCCATACGCTGAAAGGCATGAGC | This study |
|  | CheA-R | GGTTTCTACCGGCACCATCCGCATATTAAG |  |
| *gyrA* | Gyr-F | GAGACGCACTGAAATCGTGA | This study |
|  | Gyr-R | GCCGGGAGACGTTTAACATA |  |
|  | GyrA-F | CAGTCAGGAAATGCGGACATCCTT | This study |
|  | GyrA-R | CAAGATAATGCTCCAGACATTGTT |  |
| *rap* | Rap-F | AGGACATGGAAGAGGACCAA | This study |
|  | Rap-R | GTCCGGTCCCTTCAGATTTT |  |
|  | Rap1-F | ATACGAATTCATTATCGTTGCGGCATGTCG | This study |
|  | Rap1-R | ATTAGGATCCTCTAAGAGTCCGCCCCATT |  |
| *rep* | Rep-F | CATAGAATTCGAGGACTAGCATCAGAAGGAGT | This study |
|  | Rep-R | ATTAGGATCCTGAAGTCCAAGCCTTTCCG |  |
| *srfA* | Srf-F | ATTGTTTACGGTGGCTCTGG | Debois et al. (2015) |
|  | Srf-R | CGCTGCGATAGTCAAAATCA |  |
