# Supplementary material for: Key Impact of an Uncommon Plasmid on Bacillus amyloliquefaciens subsp. plantarum S499 Developmental Traits and Lipopeptide Production
Source: Front Microbiol. 2017 Jan 19;8:17. doi: 10.3389/fmicb.2017.00017 (PMC5243856; doi:10.3389/fmicb.2017.00017)
Supplement: Supplementary file 4 [file Table4.DOCX]

**Table S4. Genes present in the *Bacillus amyloliquefaciens* subsp. *plantarum* S499 genome not shared by the FZB42 genome and vice versa.** Unique CDS were identified using the sequence based comparison tool on SEED Viewer version 2.0 (Overbeek et al., 2005) and verified with NCBI annotations (http://www.ncbi.nlm.nih.gov) on S499 (CP014700) and FZB42 (CP000560) genomes.

| **S499 specific genes** | | **FZB42 specific genes** | |
| --- | --- | --- | --- |
| **Locus tag** | **Function** | **Locus tag** | **Function** |
| AS588_RS00475 | ABC transporter, permease protein | RBAM_RS00685 | 30S ribosomal protein S12 |
| AS588_RS00490 | hypothetical protein | RBAM_RS00710 | 30S ribosomal protein S10 |
| AS588_RS00500 | tRNA-guanine transglycosylase (EC 2.4.2.29) | RBAM_RS00735 | 30S ribosomal protein S19 |
| AS588_RS00505 | putative aminoglycoside 6-adenylyltansferase | RBAM_RS00740 | 50S ribosomal protein L22 |
| AS588_RS00730 | collagen adhesion protein | RBAM_RS00745 | 30S ribosomal protein S3 |
| AS588_RS00735 | sortase A, LPXTG specific | RBAM_RS00765 | 50S ribosomal protein L14 |
| AS588_RS03020 | phage protein | RBAM_RS00830 | 50S ribosomal protein L36 |
| AS588_RS03025 | hypothetical protein | RBAM_RS00840 | 30S ribosomal protein S11 |
| AS588_RS03040 | phage replication initiation | RBAM_RS00850 | 50S ribosomal protein L17 |
| AS588_RS03045 | helicase loader DnaI | RBAM_RS00880 | 30S ribosomal protein S9 |
| AS588_RS03050 | hypothetical protein | RBAM_RS01165 | membrane protein |
| AS588_RS03060 | hypothetical protein | RBAM_RS01170 | hypothetical protein |
| AS588_RS03065 | hypothetical protein | RBAM_RS01175 | hypothetical protein |
| AS588_RS03070 | hypothetical protein | RBAM_RS01205 | acriflavin resistance protein |
| AS588_RS03075 | hypothetical protein | RBAM_RS01865 | hypothetical protein |
| AS588_RS03080 | C-5 cytosine-specific DNA methylase family protein | RBAM_RS02315 | hypothetical protein |
| AS588_RS03085 | methyltransferase | RBAM_RS02595 | ester cyclase |
| AS588_RS03090 | hypothetical protein | RBAM_RS02600 | hypothetical protein |
| AS588_RS03095 | dimeric dUTPase (EC 3.6.1.23) | RBAM_RS02650 | TetR family transcriptional regulator |
| AS588_RS03105 | hypothetical protein | RBAM_RS02670 | transcriptional regulator |
| AS588_RS03115 | phage-related protein | RBAM_RS02685 | CarD family transcriptional regulator |
| AS588_RS03125 | hypothetical protein | RBAM_RS02795 | hypothetical protein |
| AS588_RS03135 | hypothetical protein | RBAM_RS02805 | hypothetical protein |
| AS588_RS03145 | phage terminase-like protein, small subunit | RBAM_RS02830 | hypothetical protein |
| AS588_RS03150 | phage terminase, large subunit | RBAM_RS02890 | SAM-dependent methyltransferase |
| AS588_RS03155 | phage portal protein | RBAM_RS02905 | TVP38/TMEM64 family protein |
| AS588_RS03160 | phage head maturation protease | RBAM_RS02920 | DUF6 transmembrane transporter |
| AS588_RS03165 | phage major capsid protein | RBAM_RS03570 | type I restriction-modification system, DNA-methyltransferase subunit M |
| AS588_RS03170 | phage tail fiber protein | RBAM_RS03575 | type I restriction-modification system, DNA-methyltransferase subunit S |
| AS588_RS03175 | hypothetical phagelike protein | RBAM_RS03580 | type I restriction-modification system, DNA-methyltransferase subunit R |
| AS588_RS03180 | FIG01228293: hypothetical protein | RBAM_RS03585 | hypothetical protein |
| AS588_RS03185 | FIG01225884: hypothetical protein | RBAM_RS03590 | McrA protein |
| AS588_RS03190 | FIG01229968: hypothetical protein | RBAM_RS03620 | RNA-binding protein, RRM domain |
| AS588_RS03195 | phage major tail protein | RBAM_RS03700 | membrane protein, putative |
| AS588_RS03200 | FIG01246408: hypothetical protein | RBAM_RS03725 | putative hydroxylase |
| AS588_RS03205 | phage tail length tape-measure protein | RBAM_RS03730 | hypothetical protein |
| AS588_RS03210 | putative tail or base plate protein gp17 [Bacteriophage A118] | RBAM_RS03735 | plantazolicin synthase D |
| AS588_RS03230 | phage protein | RBAM_RS03740 | dehydrogenase |
| AS588_RS03235 | phage protein | RBAM_RS03745 | caax amino protease family |
| AS588_RS03250 | hypothetical protein | RBAM_RS03750 | SAM-dependent methyltransferase |
| AS588_RS03255 | hypothetical protein | RBAM_RS03885 | collagen like triple helix with GXT repeats |
| AS588_RS03260 | hypothetical protein | RBAM_RS04120 | membrane protein |
| AS588_RS03330 | hypothetical protein | RBAM_RS04525 | hypothetical protein |
| AS588_RS05315 | hypothetical protein | RBAM_RS04720 | metallophosphatase |
| AS588_RS06535 | hypothetical protein | RBAM_RS05095 | hypothetical protein |
| AS588_RS06555 | transcriptional regulator | RBAM_RS05320 | mep operon protein MepB |
| AS588_RS06565 | hypothetical protein | RBAM_RS05540 | cytochrome P450 |
| AS588_RS06685 | DnaJ-class molecular chaperone CbpA | RBAM_RS05910 | sporulation protein YjcZ |
| AS588_RS08085 | sporulation protein YjcZ | RBAM_RS05990 | hypothetical protein |
| AS588_RS08315 | hypothetical protein | RBAM_RS05995 | hypothetical protein |
| AS588_RS08915 | FIG01238565: hypothetical protein | RBAM_RS06000 | DUF3037 domain-containing protein |
| AS588_RS08920 | phage-like element PBSX protein xkdU | RBAM_RS06030 | hypothetical protein |
| AS588_RS08925 | phage baseplate | RBAM_RS06105 | Kelch repeat protein |
| AS588_RS08930 | phage-like element PBSX protein xkdS | RBAM_RS06110 | hypothetical protein |
| AS588_RS08935 | FIG01234021: hypothetical protein | RBAM_RS07145 | sporulation protein YjcZ |
| AS588_RS08940 | phage-like element PBSX protein xkdQ | RBAM_RS07155 | hypothetical protein |
| AS588_RS08945 | phage-like element PBSX protein xkdP | RBAM_RS07270 | hypothetical protein |
| AS588_RS08950 | phage tail length tape-measure protein | RBAM_RS07275 | hypothetical protein |
| AS588_RS08955 | phage-like element PBSX protein xkdN | RBAM_RS07275 | hypothetical protein |
| AS588_RS08960 | phage tail fibers | RBAM_RS07925 | 50S ribosomal protein L19 |
| AS588_RS08965 | phage-like element PBSX protein xkdK | RBAM_RS08250 | 30S ribosomal protein S15 |
| AS588_RS08970 | hypothetical protein | RBAM_RS08560 | hypothetical protein |
| AS588_RS08975 | phage-like element PBSX protein xkdJ | RBAM_RS19045 | phage integrase |
| AS588_RS08980 | Lin1275 protein | RBAM_RS08720 | YoaW |
| AS588_RS08985 | phage-like element PBSX protein xkdH | RBAM_RS08745 | DUF4944 domain-containing protein YoaO |
| AS588_RS08990 | hypothetical protein | RBAM_RS08750 | hypothetical protein |
| AS588_RS08995 | phage-like element PBSX protein xkdG | RBAM_RS08780 | hypothetical protein |
| AS588_RS09000 | FIG01230357: hypothetical protein | RBAM_RS09015 | hypothetical protein |
| AS588_RS09005 | phage-like element PBSX protein xkdE | RBAM_RS09020 | hypothetical protein |
| AS588_RS09010 | phage terminase, large subunit [SA bacteriophages 11, Mu50B] | RBAM_RS09025 | hypothetical protein |
| AS588_RS09090 | lanthionine biosynthesis protein LanM | RBAM_RS09320 | IS231-related transposase |
| AS588_RS09100 | lanthionine biosynthesis protein LanM | RBAM_RS09325 | predicted short chain dehydrogenase |
| AS588_RS09105 | hypothetical protein | RBAM_RS09370 | YoaF |
| AS588_RS09120 | hypothetical protein | RBAM_RS09840 | FAD-dependent pyridine nucleotide-disulphide oxidoreductase, GBAA2537 homolog |
| AS588_RS09125 | bacitracin ABC transporter, permease protein, putative | RBAM_RS09865 | hypothetical protein |
| AS588_RS09240 | hypothetical protein | RBAM_RS09870 | hypothetical protein |
| AS588_RS09255 | hypothetical protein | RBAM_RS09875 | site-specific recombinase |
| AS588_RS09325 | lactoylglutathione lyase and related lyases | RBAM_RS10990 | DNA-binding protein |
| AS588_RS09350 | hypothetical protein | RBAM_RS11795 | 30S ribosomal protein S21 |
| AS588_RS09370 | hypothetical protein | RBAM_RS11960 | GCN5-related N-acetyltransferase |
| AS588_RS09375 | gene 22 | RBAM_RS11985 | phosphate-starvation-inducible protein PsiE |
| AS588_RS09380 | phage minor structural protein | RBAM_RS11990 | two component system histidine kinase (EC 2.7.3.-) |
| AS588_RS09385 | phage tail protein | RBAM_RS12000 | protein of unknown function DUF418 |
| AS588_RS09390 | phage tail length tape-measure protein | RBAM_RS12005 | GCN5-related N-acetyltransferase |
| AS588_RS09395 | hypothetical protein | RBAM_RS12840 | hypothetical protein |
| AS588_RS09400 | phage protein | RBAM_RS12915 | 50S ribosomal protein L35 |
| AS588_RS09405 | phage major tail protein, TP901-1 family | RBAM_RS13270 | hypothetical protein |
| AS588_RS09410 | tail protein | RBAM_RS13275 | hypothetical protein |
| AS588_RS09415 | phage capsid and scaffold | RBAM_RS13285 | hypothetical protein |
| AS588_RS09420 | phage capsid and scaffold | RBAM_RS13295 | hypothetical protein |
| AS588_RS09430 | hypothetical protein | RBAM_RS13300 | hypothetical protein |
| AS588_RS09435 | hypothetical protein | RBAM_RS13305 | hypothetical protein |
| AS588_RS09440 | phage major capsid protein | RBAM_RS13525 | transcriptional regulator, DeoR family |
| AS588_RS09445 | phage protein | RBAM_RS13675 | predicted NRPS adenylation domain |
| AS588_RS09450 | phage minor capsid protein | RBAM_RS19050 | amino acid adenylation domain-containing protein( EC:3.1.2.14,EC:5.1.1.3 ) |
| AS588_RS09455 | portal protein, phage associated | RBAM_RS13695 | conserved domain protein |
| AS588_RS09460 | phage terminase, large subunit | RBAM_RS16640 | hypothetical protein |
| AS588_RS09465 | YqaS | RBAM_RS16650 | hypothetical protein |
| AS588_RS09475 | cysteine protease | RBAM_RS17225 | hypothetical protein |
| AS588_RS09480 | transcriptional regulator | RBAM_RS17230 | hypothetical protein |
| AS588_RS09490 | hypothetical protein | RBAM_RS17255 | hypothetical protein |
| AS588_RS09495 | DNA Methyltransferase | RBAM_RS17365 | hypothetical protein |
| AS588_RS09500 | hypothetical protein | RBAM_RS17370 | hypothetical protein |
| AS588_RS09505 | hypothetical protein | RBAM_RS17375 | hypothetical protein |
| AS588_RS09525 | hypothetical protein | RBAM_RS17380 | hypothetical protein |
| AS588_RS09535 | prophage Lp1 protein 19 | RBAM_RS17485 | protein liaG |
| AS588_RS09540 | hypothetical protein | RBAM_RS17490 | bacitracin transport permease protein BCRB |
| AS588_RS09545 | hypothetical protein | RBAM_RS17810 | hypothetical protein |
| AS588_RS09555 | chain A, Nmr Structure Of Bacillus Subtilis Protein Yqai, Northeast Structural Genomics Target Sr450 | RBAM_RS18100 | hypothetical protein |
| AS588_RS09560 | DNA, complete sequence | RBAM_RS18305 | MrsG |
| AS588_RS09565 | transcriptional regulator | RBAM_RS18310 | lantibiotic ABC transporter permease |
| AS588_RS09570 | phage regulatory protein | RBAM_RS18410 | hypothetical protein |
| AS588_RS09575 | DNA-binding protein | RBAM_RS18465 | hypothetical protein |
| AS588_RS09580 | transcriptional regulator | RBAM_RS19055 | hypothetical protein |
| AS588_RS09590 | hypothetical protein | RBAM_RS19060 | resolvase domain-containing protein |
| AS588_RS09600 | phage integrase | RBAM_RS18520 | hypothetical protein |
| AS588_RS09670 | sporulation protein YjcZ | RBAM_RS18530 | hypothetical protein |
| AS588_RS10115 | hypothetical protein | RBAM_RS18535 | hypothetical protein |
| AS588_RS10325 | hypothetical protein | RBAM_RS18660 | hypothetical protein |
| AS588_RS11480 | hypothetical protein | RBAM_RS18815 | transporter, LysE family |
| AS588_RS11485 | hypothetical protein | RBAM_RS18890 | YnaF |
| AS588_RS11525 | MFS transporter, tetracycline resistance protein | RBAM_RS18895 | response regulator aspartate phosphatase |
| AS588_RS11550 | serine transporter | RBAM_RS18945 | 30S ribosomal protein S18 |
| AS588_RS11645 | hypothetical protein |  |  |
| AS588_RS11685 | hypothetical protein |  |  |
| AS588_RS11690 | FRG domain-containing protein |  |  |
| AS588_RS11700 | hypothetical protein |  |  |
| AS588_RS11710 | FtsK/SpoIIIE family protein |  |  |
| AS588_RS11975 | hypothetical protein |  |  |
| AS588_RS12450 | hypothetical protein |  |  |
| AS588_RS13030 | adenine-specific DNA modification methyltransferase |  |  |
| AS588_RS13035 | hypothetical protein |  |  |
| AS588_RS13045 | chromosome segregation ATPase |  |  |
| AS588_RS15260 | SAM-dependent methyltransferase |  |  |
| AS588_RS15340 | hypothetical protein |  |  |
| AS588_RS15925 | hypothetical protein |  |  |
| AS588_RS16115 | FIG01238735: hypothetical protein |  |  |
| AS588_RS16285 | hypothetical protein |  |  |
| AS588_RS16730 | hypothetical protein |  |  |
| AS588_RS16755 | FIG01242153: hypothetical protein |  |  |
| AS588_RS16760 | NAD(P)H-dependent oxidoreductase |  |  |
| AS588_RS16780 | hypothetical protein |  |  |
| AS588_RS16795 | hypothetical Cytosolic Protein |  |  |
| AS588_RS16995 | hypothetical protein |  |  |
| AS588_RS17325 | respiratory nitrate reductase alpha chain (EC 1.7.99.4) |  |  |
| AS588_RS17365 | phosphatase |  |  |
| AS588_RS17375 | pXO1-41 |  |  |
| AS588_RS17380 | hypothetical protein |  |  |
| AS588_RS17440 | hypothetical protein |  |  |
| AS588_RS17460 | hypothetical protein |  |  |
| AS588_RS18150 | hypothetical protein |  |  |
| AS588_RS18155 | hypothetical protein |  |  |
| AS588_RS18160 | hypothetical protein |  |  |
| AS588_RS18165 | hypothetical protein |  |  |
| AS588_RS18170 | hypothetical protein |  |  |
| AS588_RS18190 | hypothetical protein |  |  |
| AS588_RS18410 | hypothetical protein |  |  |
| AS588_RS18525 | fructose-1,6-bisphosphatase, Bacillus type (EC 3.1.3.11) |  |  |
| AS588_RS18530 | superfamily I DNA/RNA helicase protein |  |  |
| AS588_RS18535 | FIG01240834: hypothetical protein |  |  |
| AS588_RS18540 | nucleotide pyrophosphohydrolase |  |  |
| AS588_RS18555 | DEAD-like helicase |  |  |
| AS588_RS18570 | hypothetical protein |  |  |
| AS588_RS18615 | FIG038982: hypothetical protein |  |  |
| AS588_RS18695 | hypothetical protein |  |  |
| AS588_RS18865 | LysR family transcriptional regulator |  |  |
| AS588_RS18920 | transcriptional regulator, Cro/CI family |  |  |
| AS588_19070 | Rep protein |  |  |
| AS588_19090 | mobilization protein |  |  |
