# Supplementary material for: Key Impact of an Uncommon Plasmid on Bacillus amyloliquefaciens subsp. plantarum S499 Developmental Traits and Lipopeptide Production
Source: Front Microbiol. 2017 Jan 19;8:17. doi: 10.3389/fmicb.2017.00017 (PMC5243856; doi:10.3389/fmicb.2017.00017)
Supplement: Supplementary file 5 [file DataSheet1.DOCX]

**Supplementary Data 1. Alignment of 16SrDNA.** Sequences were aligned with EMBOSS Needle Pairwise Sequence Alignment tools (http://www.ebi.ac.uk/Tools/psa/).

#=======================================

#

# Aligned_sequences: 2

# 1: S499_16S

# 2: S499P-_16S

# Matrix: EDNAFULL

# Gap_penalty: 10.0

# Extend_penalty: 0.5

#

# Length: 1399

# Identity: 1399/1399 (100.0%)

# Similarity: 1399/1399 (100.0%)

# Gaps: 0/1399 ( 0.0%)

# Score: 6995.0

#

#

#=======================================

S499_16S 1 TCGAGCGGACAGATGGGAGCTTGCTCCCTGATGTTAGCGGCGGACGGGTG 50

||||||||||||||||||||||||||||||||||||||||||||||||||

S499P-_16S 1 TCGAGCGGACAGATGGGAGCTTGCTCCCTGATGTTAGCGGCGGACGGGTG 50

S499_16S 51 AGTAACACGTGGGTAACCTGCCTGTAAGACTGGGATAACTCCGGGAAACC 100

||||||||||||||||||||||||||||||||||||||||||||||||||

S499P-_16S 51 AGTAACACGTGGGTAACCTGCCTGTAAGACTGGGATAACTCCGGGAAACC 100

S499_16S 101 GGGGCTAATACCGGATGGTTGTCTGAACCGCATGGTTCAGACATAAAAGG 150

||||||||||||||||||||||||||||||||||||||||||||||||||

S499P-_16S 101 GGGGCTAATACCGGATGGTTGTCTGAACCGCATGGTTCAGACATAAAAGG 150

S499_16S 151 TGGCTTCGGCTACCACTTACAGATGGACCCGCGGCGCATTAGCTAGTTGG 200

||||||||||||||||||||||||||||||||||||||||||||||||||

S499P-_16S 151 TGGCTTCGGCTACCACTTACAGATGGACCCGCGGCGCATTAGCTAGTTGG 200

S499_16S 201 TGAGGTAACGGCTCACCAAGGCGACGATGCGTAGCCGACCTGAGAGGGTG 250

||||||||||||||||||||||||||||||||||||||||||||||||||

S499P-_16S 201 TGAGGTAACGGCTCACCAAGGCGACGATGCGTAGCCGACCTGAGAGGGTG 250

S499_16S 251 ATCGGCCACACTGGGACTGAGACACGGCCCAGACTCCTACGGGAGGCAGC 300

||||||||||||||||||||||||||||||||||||||||||||||||||

S499P-_16S 251 ATCGGCCACACTGGGACTGAGACACGGCCCAGACTCCTACGGGAGGCAGC 300

S499_16S 301 AGTAGGGAATCTTCCGCAATGGACGAAAGTCTGACGGAGCAACGCCGCGT 350

||||||||||||||||||||||||||||||||||||||||||||||||||

S499P-_16S 301 AGTAGGGAATCTTCCGCAATGGACGAAAGTCTGACGGAGCAACGCCGCGT 350

S499_16S 351 GAGTGATGAAGGTTTTCGGATCGTAAAGCTCTGTTGTTAGGGAAGAACAA 400

||||||||||||||||||||||||||||||||||||||||||||||||||

S499P-_16S 351 GAGTGATGAAGGTTTTCGGATCGTAAAGCTCTGTTGTTAGGGAAGAACAA 400

S499_16S 401 GTGCCGTTCAAATAGGGCGGCACCTTGACGGTACCTAACCAGAAAGCCAC 450

||||||||||||||||||||||||||||||||||||||||||||||||||

S499P-_16S 401 GTGCCGTTCAAATAGGGCGGCACCTTGACGGTACCTAACCAGAAAGCCAC 450

S499_16S 451 GGCTAACTACGTGCCAGCAGCCGCGGTAATACGTAGGTGGCAAGCGTTGT 500

||||||||||||||||||||||||||||||||||||||||||||||||||

S499P-_16S 451 GGCTAACTACGTGCCAGCAGCCGCGGTAATACGTAGGTGGCAAGCGTTGT 500

S499_16S 501 CCGGAATTATTGGGCGTAAAGGGCTCGCAGGCGGTTTCTTAAGTCTGATG 550

||||||||||||||||||||||||||||||||||||||||||||||||||

S499P-_16S 501 CCGGAATTATTGGGCGTAAAGGGCTCGCAGGCGGTTTCTTAAGTCTGATG 550

S499_16S 551 TGAAAGCCCCCGGCTCAACCGGGGAGGGTCATTGGAAACTGGGGAACTTG 600

||||||||||||||||||||||||||||||||||||||||||||||||||

S499P-_16S 551 TGAAAGCCCCCGGCTCAACCGGGGAGGGTCATTGGAAACTGGGGAACTTG 600

S499_16S 601 AGTGCAGAAGAGGAGAGTGGAATTCCACGTGTAGCGGTGAAATGCGTAGA 650

||||||||||||||||||||||||||||||||||||||||||||||||||

S499P-_16S 601 AGTGCAGAAGAGGAGAGTGGAATTCCACGTGTAGCGGTGAAATGCGTAGA 650

S499_16S 651 GATGTGGAGGAACACCAGTGGCGAAGGCGACTCTCTGGTCTGTAACTGAC 700

||||||||||||||||||||||||||||||||||||||||||||||||||

S499P-_16S 651 GATGTGGAGGAACACCAGTGGCGAAGGCGACTCTCTGGTCTGTAACTGAC 700

S499_16S 701 GCTGAGGAGCGAAAGCGTGGGGAGCGAACAGGATTAGATACCCTGGTAGT 750

||||||||||||||||||||||||||||||||||||||||||||||||||

S499P-_16S 701 GCTGAGGAGCGAAAGCGTGGGGAGCGAACAGGATTAGATACCCTGGTAGT 750

S499_16S 751 CCACGCCGTAAACGATGAGTGCTAAGTGTTAGGGGGTTTCCGCCCCTTAG 800

||||||||||||||||||||||||||||||||||||||||||||||||||

S499P-_16S 751 CCACGCCGTAAACGATGAGTGCTAAGTGTTAGGGGGTTTCCGCCCCTTAG 800

S499_16S 801 TGCTGCAGCTAACGCATTAAGCACTCCGCCTGGGGAGTACGGTCGCAAGA 850

||||||||||||||||||||||||||||||||||||||||||||||||||

S499P-_16S 801 TGCTGCAGCTAACGCATTAAGCACTCCGCCTGGGGAGTACGGTCGCAAGA 850

S499_16S 851 CTGAAACTCAAAGGAATTGACGGGGGCCCGCACAAGCGGTGGAGCATGTG 900

||||||||||||||||||||||||||||||||||||||||||||||||||

S499P-_16S 851 CTGAAACTCAAAGGAATTGACGGGGGCCCGCACAAGCGGTGGAGCATGTG 900

S499_16S 901 GTTTAATTTGAAGCAACGCGAAGAACCTTACCAGGTCTTGACATCCTCTG 950

||||||||||||||||||||||||||||||||||||||||||||||||||

S499P-_16S 901 GTTTAATTTGAAGCAACGCGAAGAACCTTACCAGGTCTTGACATCCTCTG 950

S499_16S 951 ACAATCCTAGAGATAGGACGTCCCCTTCGGGGGCAGAGTGACAGGTGGTG 1000

||||||||||||||||||||||||||||||||||||||||||||||||||

S499P-_16S 951 ACAATCCTAGAGATAGGACGTCCCCTTCGGGGGCAGAGTGACAGGTGGTG 1000

S499_16S 1001 CATGGTTGTCGTCAGCTCGTGTCGTGAGATGTTGGGTTAAGTCCCGCAAC 1050

||||||||||||||||||||||||||||||||||||||||||||||||||

S499P-_16S 1001 CATGGTTGTCGTCAGCTCGTGTCGTGAGATGTTGGGTTAAGTCCCGCAAC 1050

S499_16S 1051 GAGCGCAACCCTTGATCTTAGTTGCCAGCATTCAGTTGGGCACTCTAAGG 1100

||||||||||||||||||||||||||||||||||||||||||||||||||

S499P-_16S 1051 GAGCGCAACCCTTGATCTTAGTTGCCAGCATTCAGTTGGGCACTCTAAGG 1100

S499_16S 1101 TGACTGCCGGTGACAAACCGGAGGAAGGTGGGGATGACGTCAAATCATCA 1150

||||||||||||||||||||||||||||||||||||||||||||||||||

S499P-_16S 1101 TGACTGCCGGTGACAAACCGGAGGAAGGTGGGGATGACGTCAAATCATCA 1150

S499_16S 1151 TGCCCCCTATGACCTGGGCTACACACGTGCTACAATGGACAGAACAAAGG 1200

||||||||||||||||||||||||||||||||||||||||||||||||||

S499P-_16S 1151 TGCCCCCTATGACCTGGGCTACACACGTGCTACAATGGACAGAACAAAGG 1200

S499_16S 1201 GCAGCGAAACCGCGAGGTTAAGCCAATCCCACAAATCTGTTCTCAGTTCG 1250

||||||||||||||||||||||||||||||||||||||||||||||||||

S499P-_16S 1201 GCAGCGAAACCGCGAGGTTAAGCCAATCCCACAAATCTGTTCTCAGTTCG 1250

S499_16S 1251 GATCGCAGTCTGCAACTCGACTGCGTGAAGCTGGAATCGCTAGTAATCGC 1300

||||||||||||||||||||||||||||||||||||||||||||||||||

S499P-_16S 1251 GATCGCAGTCTGCAACTCGACTGCGTGAAGCTGGAATCGCTAGTAATCGC 1300

S499_16S 1301 GGATCAGCATGCCGCGGTGAATACGTTCCCGGGCCTTGTACACACCGCCC 1350

||||||||||||||||||||||||||||||||||||||||||||||||||

S499P-_16S 1301 GGATCAGCATGCCGCGGTGAATACGTTCCCGGGCCTTGTACACACCGCCC 1350

S499_16S 1351 GTCACACCACGAGAGTTTGTAACACCCGAAGTCGGTGAGGTAACCTTTA 1399

|||||||||||||||||||||||||||||||||||||||||||||||||

S499P-_16S 1351 GTCACACCACGAGAGTTTGTAACACCCGAAGTCGGTGAGGTAACCTTTA 1399

#---------------------------------------

#---------------------------------------

**Supplementary Data 2. Alignment of partial *gyrA* gene.** Sequences were aligned with EMBOSS Needle Pairwise Sequence Alignment tools (http://www.ebi.ac.uk/Tools/psa/).

#=======================================

#

# Aligned_sequences: 2

# 1: S499_GyrA

# 2: S499P-_GyrA

# Matrix: EDNAFULL

# Gap_penalty: 10.0

# Extend_penalty: 0.5

#

# Length: 925

# Identity: 925/925 (100.0%)

# Similarity: 925/925 (100.0%)

# Gaps: 0/925 ( 0.0%)

# Score: 4625.0

#

#

#=======================================

S499_GyrA 1 ATCCCGGGCGCTTCCGGATGTGCGTGACGGTCTGAAGCCGGTTCACAGAC 50

||||||||||||||||||||||||||||||||||||||||||||||||||

S499P-_GyrA 1 ATCCCGGGCGCTTCCGGATGTGCGTGACGGTCTGAAGCCGGTTCACAGAC 50

S499_GyrA 51 GGATTTTGTACGCAATGAATGATTTAGGCATGACCAGTGACAAACCATAT 100

||||||||||||||||||||||||||||||||||||||||||||||||||

S499P-_GyrA 51 GGATTTTGTACGCAATGAATGATTTAGGCATGACCAGTGACAAACCATAT 100

S499_GyrA 101 AAAAAATCTGCCCGTATCGTCGGTGAAGTTATCGGTAAGTACCACCCGCA 150

||||||||||||||||||||||||||||||||||||||||||||||||||

S499P-_GyrA 101 AAAAAATCTGCCCGTATCGTCGGTGAAGTTATCGGTAAGTACCACCCGCA 150

S499_GyrA 151 CGGTGACTCAGCGGTTTACGAATCAATGGTCAGAATGGCGCAGGATTTTA 200

||||||||||||||||||||||||||||||||||||||||||||||||||

S499P-_GyrA 151 CGGTGACTCAGCGGTTTACGAATCAATGGTCAGAATGGCGCAGGATTTTA 200

S499_GyrA 201 ACTACCGCTACATGCTTGTTGACGGACACGGCAACTTCGGTTCGGTTGAC 250

||||||||||||||||||||||||||||||||||||||||||||||||||

S499P-_GyrA 201 ACTACCGCTACATGCTTGTTGACGGACACGGCAACTTCGGTTCGGTTGAC 250

S499_GyrA 251 GGCGACTCAGCGGCCGCGATGCGTTACACAGAAGCGAGAATGTCAAAAAT 300

||||||||||||||||||||||||||||||||||||||||||||||||||

S499P-_GyrA 251 GGCGACTCAGCGGCCGCGATGCGTTACACAGAAGCGAGAATGTCAAAAAT 300

S499_GyrA 301 CGCAATGGAAATTCTGCGTGACATTACGAAAGACACGATTGACTATCAAG 350

||||||||||||||||||||||||||||||||||||||||||||||||||

S499P-_GyrA 301 CGCAATGGAAATTCTGCGTGACATTACGAAAGACACGATTGACTATCAAG 350

S499_GyrA 351 ATAACTATGACGGTTCAGAAAGAGAGCCTGCCGTCATGCCTTCGAGATTT 400

||||||||||||||||||||||||||||||||||||||||||||||||||

S499P-_GyrA 351 ATAACTATGACGGTTCAGAAAGAGAGCCTGCCGTCATGCCTTCGAGATTT 400

S499_GyrA 401 CCGAATCTGCTCGTAAACGGGGCTGCCGGTATTGCGGTCGGAATGGCGAC 450

||||||||||||||||||||||||||||||||||||||||||||||||||

S499P-_GyrA 401 CCGAATCTGCTCGTAAACGGGGCTGCCGGTATTGCGGTCGGAATGGCGAC 450

S499_GyrA 451 AAACATTCCCCCGCATCAGCTTGGGGAAGTCATTGAAGGCGTGCTTGCCG 500

||||||||||||||||||||||||||||||||||||||||||||||||||

S499P-_GyrA 451 AAACATTCCCCCGCATCAGCTTGGGGAAGTCATTGAAGGCGTGCTTGCCG 500

S499_GyrA 501 TAAGTGAGAATCCTGAGATTACAAACCAGGAGCTGATGGAATACATCCCG 550

||||||||||||||||||||||||||||||||||||||||||||||||||

S499P-_GyrA 501 TAAGTGAGAATCCTGAGATTACAAACCAGGAGCTGATGGAATACATCCCG 550

S499_GyrA 551 GGCCCGGATTTTCCGACTGCAGGTCAGATTTTGGGCCGGAGCGGCATCCG 600

||||||||||||||||||||||||||||||||||||||||||||||||||

S499P-_GyrA 551 GGCCCGGATTTTCCGACTGCAGGTCAGATTTTGGGCCGGAGCGGCATCCG 600

S499_GyrA 601 CAAGGCATATGAATCCGGACGGGGATCAATCACGATCCGGGCTAAGGCTG 650

||||||||||||||||||||||||||||||||||||||||||||||||||

S499P-_GyrA 601 CAAGGCATATGAATCCGGACGGGGATCAATCACGATCCGGGCTAAGGCTG 650

S499_GyrA 651 AAATCGAAGAGACTTCATCGGGAAAAGAAAGAATTATTGTCACGGAACTT 700

||||||||||||||||||||||||||||||||||||||||||||||||||

S499P-_GyrA 651 AAATCGAAGAGACTTCATCGGGAAAAGAAAGAATTATTGTCACGGAACTT 700

S499_GyrA 701 CCTTATCAGGTGAACAAAGCGAGATTAATTGAAAAAATCGCGGATCTTGT 750

||||||||||||||||||||||||||||||||||||||||||||||||||

S499P-_GyrA 701 CCTTATCAGGTGAACAAAGCGAGATTAATTGAAAAAATCGCGGATCTTGT 750

S499_GyrA 751 CCGAGACAAAAAAATCGAAGGAATTACCGATCTGCGAGACGAATCCGACC 800

||||||||||||||||||||||||||||||||||||||||||||||||||

S499P-_GyrA 751 CCGAGACAAAAAAATCGAAGGAATTACCGATCTGCGAGACGAATCCGACC 800

S499_GyrA 801 GTAACGGAATGAGAATCGTCATTGAGATCCGCCGTGACGCCAATGCTCAC 850

||||||||||||||||||||||||||||||||||||||||||||||||||

S499P-_GyrA 801 GTAACGGAATGAGAATCGTCATTGAGATCCGCCGTGACGCCAATGCTCAC 850

S499_GyrA 851 GTCATTTTGAATAACCTGTACAAACAAACGGCCCTGCAGACGTCTTTCGG 900

||||||||||||||||||||||||||||||||||||||||||||||||||

S499P-_GyrA 851 GTCATTTTGAATAACCTGTACAAACAAACGGCCCTGCAGACGTCTTTCGG 900

S499_GyrA 901 AATCAACCTGCTGGCGCTCGTTGAC 925

|||||||||||||||||||||||||

S499P-_GyrA 901 AATCAACCTGCTGGCGCTCGTTGAC 925

#---------------------------------------

#---------------------------------------

**Supplementary Data 3. Alignment of partial *cheA* gene.** Sequences were aligned with EMBOSS Needle Pairwise Sequence Alignment tools (http://www.ebi.ac.uk/Tools/psa/).

#=======================================

#

# Aligned_sequences: 2

# 1: S499_CheA

# 2: S499P-_CheA

# Matrix: EDNAFULL

# Gap_penalty: 10.0

# Extend_penalty: 0.5

#

# Length: 850

# Identity: 850/850 (100.0%)

# Similarity: 850/850 (100.0%)

# Gaps: 0/850 ( 0.0%)

# Score: 4250.0

#

#

#=======================================

S499_CheA 1 ATCTGGCGCATTTAACCCACCTGATGGAAAATGTGCTGGACGCCATCCGC 50

||||||||||||||||||||||||||||||||||||||||||||||||||

S499P-_CheA 1 ATCTGGCGCATTTAACCCACCTGATGGAAAATGTGCTGGACGCCATCCGC 50

S499_CheA 51 AACGGAGAAATGCCCGTTACATCGGATTGGCTGGACGTGCTGTTTGAAGC 100

||||||||||||||||||||||||||||||||||||||||||||||||||

S499P-_CheA 51 AACGGAGAAATGCCCGTTACATCGGATTGGCTGGACGTGCTGTTTGAAGC 100

S499_CheA 101 GCTTGATCATCTTGAAGAGATGGTGCAGTCCATTATTGACGGAGGAGACG 150

||||||||||||||||||||||||||||||||||||||||||||||||||

S499P-_CheA 101 GCTTGATCATCTTGAAGAGATGGTGCAGTCCATTATTGACGGAGGAGACG 150

S499_CheA 151 GCAAACGTGATATTTCTGAAGTAAGTGCGAAGCTCGACGTAAATGCGGTG 200

||||||||||||||||||||||||||||||||||||||||||||||||||

S499P-_CheA 151 GCAAACGTGATATTTCTGAAGTAAGTGCGAAGCTCGACGTAAATGCGGTG 200

S499_CheA 201 CATGAGACTGCGGCTTCAGCCGAAACAGCAGAACCGCCGGCTTCAAAACA 250

||||||||||||||||||||||||||||||||||||||||||||||||||

S499P-_CheA 201 CATGAGACTGCGGCTTCAGCCGAAACAGCAGAACCGCCGGCTTCAAAACA 250

S499_CheA 251 ACAGACTTCAACTGAATGGAATTATGATGAGTTCGAACGGACTGTTATTG 300

||||||||||||||||||||||||||||||||||||||||||||||||||

S499P-_CheA 251 ACAGACTTCAACTGAATGGAATTATGATGAGTTCGAACGGACTGTTATTG 300

S499_CheA 301 AAGAGGCGGAAGAGCAAGGATTCAGCCGCTATGAAATAACGGTTTCCCTG 350

||||||||||||||||||||||||||||||||||||||||||||||||||

S499P-_CheA 301 AAGAGGCGGAAGAGCAAGGATTCAGCCGCTATGAAATAACGGTTTCCCTG 350

S499_CheA 351 AATGAAAGCTGCATGCTCAAAGCCGTGCGCGTGTACATGATATTTGAAAA 400

||||||||||||||||||||||||||||||||||||||||||||||||||

S499P-_CheA 351 AATGAAAGCTGCATGCTCAAAGCCGTGCGCGTGTACATGATATTTGAAAA 400

S499_CheA 401 GCTGAATGAAGCCGGGGAAGTCGCAAAAACGATACCTGCCGCGGAAGTGC 450

||||||||||||||||||||||||||||||||||||||||||||||||||

S499P-_CheA 401 GCTGAATGAAGCCGGGGAAGTCGCAAAAACGATACCTGCCGCGGAAGTGC 450

S499_CheA 451 TTGAGACGGAAGATTTCGGAACGGATTTTCAGGTGTGTTTCTTGACAAAG 500

||||||||||||||||||||||||||||||||||||||||||||||||||

S499P-_CheA 451 TTGAGACGGAAGATTTCGGAACGGATTTTCAGGTGTGTTTCTTGACAAAG 500

S499_CheA 501 CAGCCTGCTGAAGAAATTAAAGAACTCATCAGCGGCATTTCAGAAGTGGA 550

||||||||||||||||||||||||||||||||||||||||||||||||||

S499P-_CheA 501 CAGCCTGCTGAAGAAATTAAAGAACTCATCAGCGGCATTTCAGAAGTGGA 550

S499_CheA 551 GAATGTCGAAATTTCTGCCGGTGCGCCGTTAAAAACGGCCGAAAAACCGC 600

||||||||||||||||||||||||||||||||||||||||||||||||||

S499P-_CheA 551 GAATGTCGAAATTTCTGCCGGTGCGCCGTTAAAAACGGCCGAAAAACCGC 600

S499_CheA 601 AAGCAGCTGAACCGGTGAAGGAAACTCCGGTTAAAAAGGCTGAAAAACAG 650

||||||||||||||||||||||||||||||||||||||||||||||||||

S499P-_CheA 601 AAGCAGCTGAACCGGTGAAGGAAACTCCGGTTAAAAAGGCTGAAAAACAG 650

S499_CheA 651 CCGAAACCGCAAGCGAAAACGGAAGAGCAGCCGAAGCATCACAGCGGCTC 700

||||||||||||||||||||||||||||||||||||||||||||||||||

S499P-_CheA 651 CCGAAACCGCAAGCGAAAACGGAAGAGCAGCCGAAGCATCACAGCGGCTC 700

S499_CheA 701 GAAAACGATTCGCGTCAACATTGAAAGACTGGATTCTTCAATGAACCTTT 750

||||||||||||||||||||||||||||||||||||||||||||||||||

S499P-_CheA 701 GAAAACGATTCGCGTCAACATTGAAAGACTGGATTCTTCAATGAACCTTT 750

S499_CheA 751 TTGAAGAACTTGTCATTGACCGCGGACGTCTTGAGCAGATCGCCAAAGAG 800

||||||||||||||||||||||||||||||||||||||||||||||||||

S499P-_CheA 751 TTGAAGAACTTGTCATTGACCGCGGACGTCTTGAGCAGATCGCCAAAGAG 800

S499_CheA 801 CTTGACCACAATGAGCTGACTGAAACCGTTGAACGCCTGACCAGAATTTC 850

||||||||||||||||||||||||||||||||||||||||||||||||||

S499P-_CheA 801 CTTGACCACAATGAGCTGACTGAAACCGTTGAACGCCTGACCAGAATTTC 850

#---------------------------------------

#---------------------------------------
