# Supplementary material for: Key Impact of an Uncommon Plasmid on Bacillus amyloliquefaciens subsp. plantarum S499 Developmental Traits and Lipopeptide Production
Source: Front Microbiol. 2017 Jan 19;8:17. doi: 10.3389/fmicb.2017.00017 (PMC5243856; doi:10.3389/fmicb.2017.00017)
Supplement: Supplementary file 6 [file Image1.PDF]

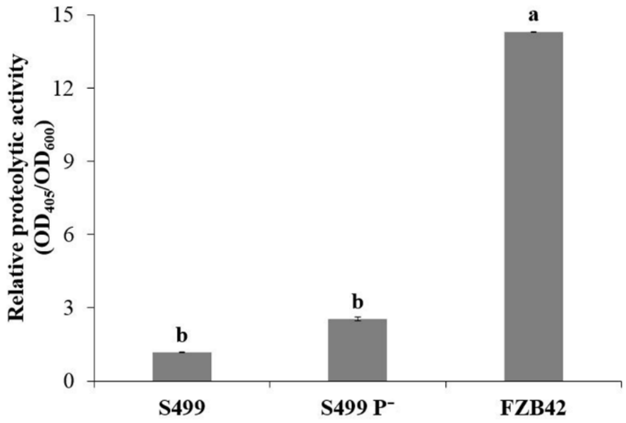

Figure S1: Extracellular proteolytic activity. Bacterial culture filtrates of *Bacillus amyloliquefaciens* subsp. *plantarum* FZB42, S499 and its plasmid-cured derivative, S499 P^-, tested with azocasein assay. Absorbance at 600nm (OD_{600}) indicates cell growth after 6 h incubation at 28°C in LB medium. Absorbance at 405nm (OD_{405}) indicates the quantity of digested casein by the extracellular protease released in culture filtrates. Average values of three replicates from one representative experiment are shown. Error bars represent standard errors. Different letters indicate significant differences according to Tukey's test ($\alpha = 0.05$). The experiment was repeated.
