# Supplementary material for: Key Impact of an Uncommon Plasmid on Bacillus amyloliquefaciens subsp. plantarum S499 Developmental Traits and Lipopeptide Production
Source: Front Microbiol. 2017 Jan 19;8:17. doi: 10.3389/fmicb.2017.00017 (PMC5243856; doi:10.3389/fmicb.2017.00017)
Supplement: Supplementary file 7 [file Image2.PDF]

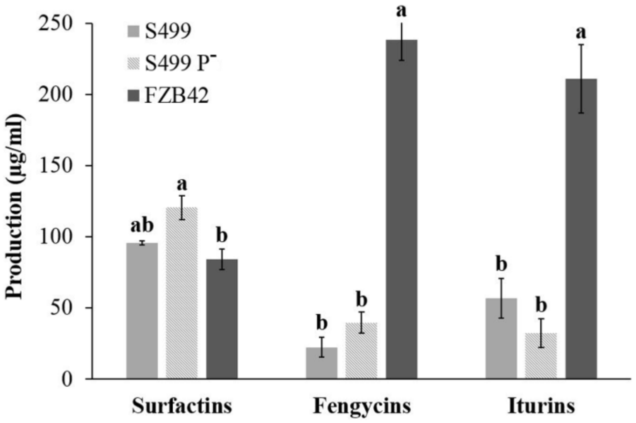

Figure S2: Production of cyclic lipopeptides. Surfactin, fengycin and iturin concentrations detected through UPLC-MS in the bacterial culture filtrates of *Bacillus amyloliquefaciens* subsp. *plantarum* FZB42, S499 and its plasmid-cured derivative, S499 P^{-}, upon 24 h growth at 28°C in LB medium. Production values correspond to the resulting averages of standardised data [Z=(X/µ)*100] from three independent experiments. Error bars represent standard errors. Different letters indicate significant differences according to Tukey's test ($\alpha = 0.05$).
