# Supplementary material for: Key Impact of an Uncommon Plasmid on Bacillus amyloliquefaciens subsp. plantarum S499 Developmental Traits and Lipopeptide Production
Source: Front Microbiol. 2017 Jan 19;8:17. doi: 10.3389/fmicb.2017.00017 (PMC5243856; doi:10.3389/fmicb.2017.00017)
Supplement: Supplementary file 8 [file Image3.PDF]

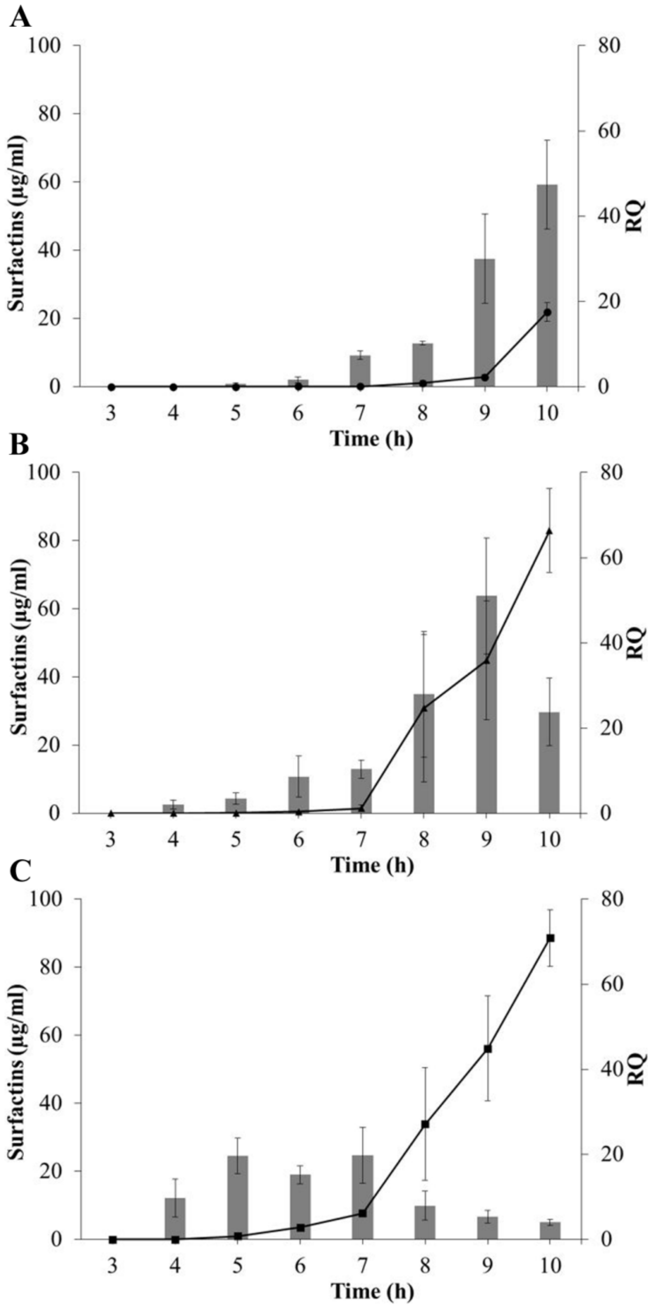

Figure S3: Surfactin production and relative quantity of *srfA* gene expression. Surfactin production (lines) and fold-increase of *srfA* gene expression compared to "time 0" (histograms) in *B. amyloliquefaciens* subsp. *plantarum* S499 **(A)**, its plasmid-cured derivative, S499 P^- **(B)** and FZB42 **(C)**. Production values correspond to the resulting averages of standardised data [Z=(X/µ)*100] from three independent experiments. Average RQ values of the three experiments are shown. Error bars represent standard errors.
